# Metastatic breast cancer cells are vulnerable to fatty acid oxidation inhibition through DDX3-DRP1-mediated mitochondrial plasticity

**DOI:** 10.1016/j.redox.2025.103845

**Published:** 2025-08-26

**Authors:** Wen-Jing Hsu, Ming-Chien Hsu, Cheng-Ying Chu, Yu-Cheng Lee, Ching-Chieh Yang, Zei-Wei Liu, Chi-Ching Lee, Yang-Sen Lin, Cheng-Wei Lin

**Affiliations:** a Graduate Institute of Medical Sciences, College of Medicine, Taipei Medical University, Taipei, Taiwan; b Department of Biochemistry and Molecular Cell Biology, School of Medicine, College of Medicine, Taipei Medical University, Taipei, Taiwan; c CRISPR Gene Targeting Core Lab, Taipei Medical University, Taipei, Taiwan; d Department of Radiation Oncology, Chi Mei Medical Center, Tainan, Taiwan; e Department of Pharmacy, Chia-Nan University of Pharmacy and Science, Tainan, Taiwan; f School of Medicine, College of Medicine, National Sun Yat-sen University, Kaohsiung, Taiwan; g Department of Food Engineering, Faculty of Engineering and Natural Sciences, Istanbul Sabahattin Zaim University, Istanbul, Turkey; h Department of Biomedical Science and Environmental Biology, Kaohsiung Medical University, Kaohsiung, Taiwan

**Keywords:** DDX3, DRP1, Mitochondrial fission, FAO, Tumor metastasis

## Abstract

Metastatic tumor cells exhibit distinct metabolic flexibility in overcoming different microenvironmental obstacles and thriving in a secondary organ; thus, metabolic vulnerabilities can potentially be targeted. It was reported that mitochondrial biogenesis and dynamics play crucial roles in disseminated tumor cells satisfying their energy demands and metabolic plasticity. However, the detailed molecular mechanism by which mitochondrial dynamics promotes tumor metastasis is still unclear. Herein, we identified that metastatic breast cancer cells exhibited increased lipid contents in mitochondria and promoted a metabolic shift towards fatty acid oxidation (FAO). The increased FAO was accompanied by promotion of mitochondrial fission. Mechanistically, we found that upregulation of DEAD-box polypeptide 3, X-linked (DDX3) promoted mitochondrial fission and facilitated FAO. Suppression of DDX3 diminished FAO and elicited mitochondrial oxidative stress in metastatic tumor cells. Moreover, DDX3 mediated dynamin-related protein 1 (DRP1) phosphorylation at S616 through collaborating with cyclin-dependent kinase 1 (CDK1). Inhibition of the DDX3-DRP1-CDK1 axis reduced cancer stemness properties and tumor metastasis. Our findings indicate that DDX3 modulates mitochondrial plasticity to drive metabolic adaptation in breast tumor metastasis. DDX3 provides a potential diagnostic biomarker and therapeutic vulnerability through which cancer metabolism can be targeted.

## Highlights

• Metastatic breast cancer cells rely on FAO.
• DDX3 enhances mitochondrial fission to support FAO in metastatic breast cancer cells.
• DDX3 cooperates with CDK1 to facilitate DRP1 phosphorylation.
• Fatty acid stimulation reinforces the DDX3-CDK1-DRP1 interaction.
• Targeting DDX3 impairs FAO and selectively suppresses metastatic traits of breast cancer cells.

## Graphical abstract

**Figure:**
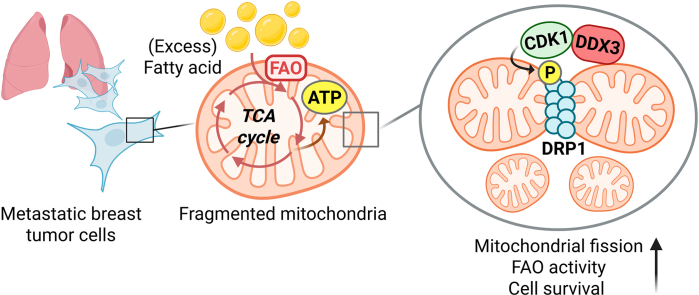

## 1 Introduction

Metastatic relapses commonly occur in cancer patients after a primary diagnosis or treatment, and they remain largely incurable [1,2]. Disseminated tumor cells with specialized metastatic characteristics can adapt, survive, colonize distal organs, and create a permissive niche by overcoming various obstacles, including nutrient limitations, oxidative stress, and other microenvironmental factors like matrix detachment-induced cell death and immune defenses [3]. Distantly metastasizing tumor cells, with stem-like features recognizing increased nutrient availability and flexibility, are responsible for late recurrences [4,5]. Therefore, understanding traits and molecular mechanisms which overcome metastatic stress has been a critical issue for developing cancer therapeutic strategies.

Metabolic reprogramming is a pivotal hallmark of cancer evolution during metastatic progression [6,7]. Metastasizing cells undergo dynamic metabolic changes in order to adapt to unfavorable and challenging microenvironments when circulating into distant organs [8]. Therefore, disseminated tumor cells display metabolic plasticity in which they utilize differential metabolites to fuel various metabolic requirements in the metastatic cascade [9]. The availability of nutrients and their associated dependencies govern the metabolic reprogramming of disseminated cancer cells [10]. Multiple metabolites and nutrients foster the invasiveness and migratory abilities of cancer cells and their survival in the circulation [4]. For instance, disseminated breast cancer cells encounter unique microenvironments, such as in the brain, a lipid-rich organ, and thus have to rewrite their metabolism [11]. Consequently, brain tropic latent metastatic breast cancer cells are known to be enriched in metabolites and genes associated with fatty acid (FA) metabolism [11,12]. Ample evidence indicates that FA uptake and metabolism can facilitate the nesting of metastasizing tumor cells in multiple organs [13,14]. Targeting the FA translocase, cluster of differentiation 36 (CD36), had no significant effects on primary tumor growth, while it dramatically suppressed metastasis in lungs and lymph nodes of tumor-bearing mice [[15], [16], [17]]. Since tumor cells at primary sites and metastatic organs exhibit metabolically distinct properties and accumulating evidence has shown that mitochondrial metabolism is preferentially utilized by metastasizing cancer cells, targeting nutrient availability and metabolic dependency in disseminated tumor cells is recognized as a potential strategy to restrict tumor progression.

Mitochondria, as cellular energy powerhouses, not only satisfy cellular bioenergetic needs but also generate metabolites which facilitate molecular and epigenomic responses, cellular metabolism, signaling transduction, and reactive oxygen species (ROS) production [18]. Mitochondria are characterized as being highly dysregulated in tumor cells, in which mitochondrial dynamics are delicately orchestrated to constantly undergo cycles of fission and fusion [19]. The mitochondrial network architecture is determined by the homeostasis between mitochondrial fission and fusion, which regulates several important cellular processes, such as proliferation, apoptosis, bioenergetic adaptation to nutrient availability or flexibility, and mitophagy [20,21]. Mitochondrial fission is controlled by dynamin-related protein 1 (DRP1, encoded by the *DNM1L* gene), a small GTPase in the dynamin family. Numbers of adapter proteins localized in the mitochondrial outer membrane, including Fis1 and MFF, function as receptors to recruit DRP1 to mitochondria, which is followed by constriction of DRP1 [22]. Furthermore, DRP1 is known to be phosphorylated at the S616 site by several kinases, including extracellular signal-regulated kinase (ERK), Rho-associated coiled-coil kinase 1 (ROCK1), Ca^2+^/calmodulin-dependent protein kinase (CamK), and cyclin-dependent kinases (CDKs), which increases its ability to facilitate mitochondrial fission [23]. Conversely, reversible phosphorylation of DRP1 at the S637 site by protein kinase A (PKA) and calcineurin is known to inhibit mitochondrial fission [24,25]. Growing evidence suggests that stem-like tumor cells exhibit highly fragmented mitochondrial networks [[26], [27], [28]]. In contrast, suppression of mitochondrial fusion was shown to promote tumorigenesis [29], emphasizing the association of mitochondrial fragmentation and cancer metastasis [30]. However, the role of mitochondrial dynamics and its mechanism which contributes to metastatic progression in breast cancer remain largely unclear.

Our previous work uncovered that DEAD-box polypeptide 3, X-linked (DDX3) expression was elevated in metastatic breast cancer, and that DDX3 mediates mitochondrial homeostasis to promote breast cancer metastasis [31]. DDX3 is an RNA helicase that plays a crucial role in regulating RNA stability and protein translation. DDX3 overexpression was associated with several types of malignancy, including breast, lung, and prostate cancers [32]. We demonstrated that DDX3 promotes mitophagy by enhancing phosphatase and tensin homolog (PTEN)-induced kinase 1 (PINK1) protein translation [31]. Intriguingly, we found that stimulation of mitochondrial stresses led to an increase of mitochondria localization of DDX3 [31]. Moreover, DDX3 expression is associated with mitochondrial dynamics, yet the underlying mechanism has not been investigated. Herein, we identified that DDX3 interacts with and promotes phosphorylation of DRP1, and as a result, DDX3 expression enhances mitochondrial fission and drives the metabolic shift towards FAO in metastatic breast cancer cells. Inhibition of DDX3 impaired mitochondrial fission and thus diminished FAO activity and suppressed the progression of breast cancer cells. We uncovered the crucial role of DDX3 by harnessing mitochondrial dynamics to facilitate metabolic reprogramming in metastatic breast tumor cells. Our study highlights the therapeutic vulnerability by targeting mitochondrial metabolism as a novel and potential strategy for treating breast cancer metastasis.

## 2 Materials and methods

### 2.1 Cell culture

The BT549 and MCF7 human breast cancer cell lines, the 4T1 mouse breast cancer cell line, and the HEK293T cell line were maintained in Dulbecco's modified Eagle medium (DMEM) supplemented with 10 % fetal bovine serum (FBS). All media were supplemented with 1 % penicillin-streptomycin, 1 % antibiotic-antimycotic, and 1 % Glutagro. All cell culture supplements were purchased from Corning Costar. All cells were cultured at 37 °C in a humidified atmosphere of 5 % CO_2_.

### 2.2 Plasmid transfection and viral infection for stable cell line establishment

The pCMV3-HA-DDX3, pCMV3-Myc-DDX3, and pCMV3-HA-DRP1 plasmids were purchased from Sino Biological. Transfection was performed with PolyJet™ In Vitro DNA Transfection Reagent (SL100688, SignaGen Laboratories) following the manufacturer's instructions as previously described [31]. Specific sequences of short hairpin (sh)RNAs against DDX3 are listed below: shhDDX3, CCGGCGGAGTGATTACGATGGCATTCTCGAGAATGCCATCGTAATCACTCCGTTTTT; and shmDDX3, CCGGGCTGTGATTCTCCACTGAAATCTCGAGATTTCAGTGGAGAATCACAGCTTTTTG.

### 2.3 Cell viability, colony formation and cell proliferation assay

Cell viability was measured using the 3-(4,5-dimethylthiazol-2-yl)-2,5-diphenyltetrazolium bromide (MTT) assay. Colony formation was assessed by crystal violet staining and quantified with ImageJ software. Cell proliferation was evaluated over a 4-day period. Detailed protocols for these assays were described in our previous study [31,33].

### 2.4 Wound-healing assay

4T1-PT and 4T1-LM cells were seeded in six-well plates and incubated overnight to form a monolayer. The following day, cells were gently scratched with a pipette tip across the center of the well. After scratching, the well was washed with medium to remove any detached cells. Cell medium was refreshed with medium containing 0.7 % FBS, and cells were treated with different inhibitors as indicated for 24 h. After incubation, cells were washed with phosphate-buffered saline (PBS) and photographed under an inverted microscope. The wound area in each condition was quantitatively evaluated using ImageJ software.

### 2.5 RNA extraction, reverse transcription (RT), and an RT-quantitative polymerase chain reaction (qPCR)

Total RNA in indicated conditions was extracted with a GENzolTM TriRNA Pure kit (GZX100, Geneaid). cDNA was synthesized with M-MLV reverse transcriptase (M5313, Promega) according to the manufacturer's instructions. cDNA was analyzed by a qPCR with GoTaq qPCR Master Mix (A6001, Promega) and gene-specific primers in a PCRmax ECO48 Real-Time qPCR system (PCRmax). Real-time PCR results were analyzed and expressed as relative expression of the threshold cycle (CT) using the ΔΔCT equation method. Sequences of specific qPCR primers used in this study are listed below: DDX3X, 5′-TGCTGGCCTAGACCTGAACT-3’ (forward) and 5′-CTTTAGTAGCTTCTCGGTTCCTT-3’ (reverse); OCT4, 5′-ATTCAGCCAAACGACCATCT-3’ (forward) and 5′-ACACTCGGACCACATCCTTC-3’ (reverse); SOX2, 5′-TACAGCATGTCCTACTCGCAG-3’ (forward) and 5′-GAGGAAGAGGTAACCACAGGG-3’ (reverse); and NANOG, 5′-GTCCCGGTCAAGAAACAGAA-3’ (forward) and 5′-TGCGTCACACCATTGCTATT-3’ (reverse).

### 2.6 Western blotting

Mitochondrion and cytosolic fractions were prepared using a mitochondrion/cytosol fractionation kit (ab65320, Abcam) as per the manufacturer's instructions. Total cellular lysates in different conditions were lysed in ice-cold RIPA buffer (50 mM Tris-HCl (pH 7.4), 150 mM NaCl, 1 mM EGTA, 1 % NP-40, and 0.025 % sodium deoxycholate) supplemented with a protease and phosphatase inhibitor cocktail (04693132001, Roche). Western blotting was performed following a standard protocol as described in our previous studies [34]. The information of antibodies are listed below: DDX3 (cat. no. GTX110614), CDK1 (cat. no. GTX50559), COXIV (cat. no. GTX114330), TOMM20 (cat. no. GTX00773), TOMM20 (SC-17764), β-actin (cat. no. GTX109639), Ki67 (cat. no. GTX16667), Myc-tag (cat. no. GTX115046), pDRP1^S616^ (cat. no. 4494), DRP1 (cat. no. 8570), MFN1 (cat. no. 14739), HA-tag (cat. no. SC-7392), peroxidase AffiniPure goat anti-mouse immunoglobulin G (IgG) (H + L) (cat. no. 115-035-003).

### 2.7 Co-immunoprecipitation (Co-IP) assay

Cells in different conditions were lysed with ice-cold mild lysis buffer (25 mM Tris-HCl (pH 7.4), 150 mM NaCl, 1 mM EDTA (pH 8), and 1 % NP-40) supplemented with a protease inhibitor cocktail. Collected supernatants were then incubated with desired antibodies and protein A/G PLUS-agarose (sc-2003, Santa Cruz) with agitation at 4 °C overnight. After overnight incubation, samples were centrifuged at 3000 rpm and 4 °C and washed three times with mild lysis buffer. Samples were then boiled with 2 × sodium dodecylsulfate (SDS) loading buffer and subjected to a Western blot assay. Primary antibodies used for the IP analysis are mentioned in the Western blotting section.

### 2.8 Mass spectrometric (MS) analysis identifying DDX3-interacting mitochondrial proteins

To identify DDX3-interacting mitochondrial proteins upon carbonylcyanide 3-chlorophenylhydrazone (CCCP) induction, HA-DDX3-overexpressing BT549 cells were treated with or without CCCP for 6 h, and the mitochondrial fractions in both conditions were purified as previously described. Next, mitochondrial fraction proteins (2 mg) were immunoprecipitated with an anti-HA antibody (2 μg), separated in 10 % SDS-polyacrylamide gel electrophoresis (PAGE), and visualized by colloidal Coomassie blue staining. Proteins in the gel were reduced and alkylated using dithiothreitol and iodoacetamide followed by trypsin digestion. Tryptic peptides were collected and desalted on a C18 SPE cartridge, and analyzed using a nano liquid chromatography-tandem MS (LC-MS/MS; Orbitrap Elite ETD; Thermo Fisher). Protein identification (SwissProt database) and data interpretation were performed using PEAKS studio 10.5 software.

### 2.9 Flow cytometry

To determine changes in the mitochondrial mass and mitochondrial superoxide after palmitic acid (PA) treatments, 10^5^ cells were seeded into 12-well plates in indicated conditions and individually incubated with 50 nM MitoBright LT Red (MT11, Dojindo) and 50 nM MitoSOX™ Red (M36008, Invitrogen). After incubation for 30 min at 37 °C, cells were washed twice with PBS and analyzed by flow cytometry (BD Biosciences). For cell apoptosis measurement, 8 × 10^4^ cells were seeded into 12-well plates in indicated conditions and treated with PA (200 μM) for 24 h. Cells were stained using an Annexin V-FITC/7-AAD Apoptosis Kit (E-CK-A212, Elabscience) as per the manufacturer's instructions and detected by flow cytometry. Apoptosis was calculated by summing up percentages of Annexin V-FITC^+^ propidium iodide (PI)^-^ (early apoptotic) and Annexin V-FITC^+^ PI^+^ (late apoptotic) cells. Data were analyzed using FACSDiva™ software.

### 2.10 Seahorse assay

The oxygen consumption rate (OCR) was measured with a Seahorse XFe24 Flux Analyzer (Seahorse Bioscience) according to the manufacture's instruction. For the FAO activity assay, the culture medium was replaced with XF basal medium (Seahorse Bioscience) supplemented with or without or both 100 μM PA and 10 μM etomoxir for 3 h, and plates were placed in a 37 °C non-CO_2_ incubator. The basal respiration, maximal respiration, ATP production, and spare respiration capacities were calculated the same as for the mito stress assay.

### 2.11 Lipidomic analysis

To compare the difference between 4T1-PT and 4T1-LM lipidomic profiles, we collected 4T1-PT and 4T1-LM cell lysates with three independent repeats. Cell samples of 300 μL in 80 % methanol (MeOH) were supplemented with 30 μL MeOH, extracted with 900 μL methyl-*tert*-butyl ether (MTBE), evenly mixed for more than 1 min, and allowed to sit at room temperature for 60 min. After adding 156 μL water, samples were centrifuged at 12,000 *g* and 4 °C for 30 min, and the supernatant was dried with nitrogen gas. Samples were redissolved in 1000 μL of an isopropyl alcohol (IPA)/acetonitrile/water (2:1:1, v/v/v) mixture, and centrifuged at 12,000 *g* and 4 °C for 30 min. The supernatant was subjected to a lipidomic analysis with ultra-high-performance LC coupled with Xevo G2XS (Waters Corp., Milford, MA, USA).

### 2.12 Tumorsphere-formation assay

To evaluate the stemness of BT549 and MCF7 cells, cells (10^3^) were seeded into ultra-low-attachment six-well microplates (Corning Costar). Cells were maintained in serum-free DMEM/F-12 supplemented with 25 ng/mL of epidermal growth factor (Z00333-1, GeneScript), 50 ng/mL basic fibroblast growth factor (1140-02-10, Biocompare), and B27 (17504044, Gibco). Cells were then allowed to grow for 7 days, and tumorspheres were observed under a light microscope. Tumorsphere sizes in different treatments were evaluated and documented.

### 2.13 Immunofluorescent (IF) staining and imaging

For confocal microscopic imaging, cells (5 × 10^4^) were seeded on glass coverslips in 12-well plates and left overnight for cell attachment. After treatment, cells were fixed in 4 % paraformaldehyde (PFA) for 10 min at room temperature. Cells were further permeabilized with 0.3 % Triton X-100 in 1 % bovine serum albumin (BSA) in PBS for 15 min on ice, and blocked with 1 % BSA in PBS at room temperature for 30 min. Desired primary antibodies were incubated with cells overnight at 4 °C, and secondary antibodies were incubated with cells at room temperature for 30 min. Cells were washed with cold PBS, and cell nuclei were then counterstained with DAPI using mounting medium (ab104139, Abcam). Photographs were taken with a confocal microscope. The primary antibodies and the secondary antibodies used for IF staining were shown in Western blotting section and listed below: CoraLite® Plus 488-conjugated DDX3 Recombinant antibody (cat. no. CL488-81903), CoraLite® Plus 647-conjugated DRP1 (C-terminal) Polyclonal antibody (cat. no. CL647-12957), Multi-rAb™ CoraLite® Plus 555-Goat Anti-Rabbit Recombinant Secondary Antibody (H + L) (cat. no. RGAR003), peroxidase AffiniPure goat anti-rabbit IgG (H + L) (cat. no. 111-035-003), goat anti-rabbit IgG antibody (DyLight488) (cat. no. GTX213110-04), and goat anti-mouse IgG antibody (DyLight594) (cat. no. GTX213111-05).

### 2.14 Immunohistochemical (IHC) staining and tissue microarray (TMA) evaluation

Formalin-fixed and paraffin-embedded 4T1-PT, 4T1-LM, and 4T1-LM shDDX3 tumor tissues were deparaffinized and rehydrated. Antigen retrieval was performed using a 10 mM citric acid-based (H-3300, Vector Laboratories) or 10 mM Tris-based unmasking buffer (H-3301-250, Vector Laboratories) and boiling for 1 h. Endogenous peroxidase activity was blocked with 4 % H_2_O_2_ in double-distilled (dd)H_2_O. Tumor tissues were then blocked with 3 % BSA in PBS and incubated with desired primary antibodies overnight at 4 °C. The next day, tissues were incubated with Signal Stain Boost IHC detection reagent (no. 8125 and no. 8114, Cell Signaling Technology) for 1 h at room temperature. Slides were washed with PBS, and staining was visualized with a 3,3′-diaminobenzidine (DAB) substrate kit (SK-4100, Vector Laboratories) and counterstained with Mayer's Hematoxylin Solution (MHS32, Sigma-Aldrich). Tissue sections were then washed twice with isopropanol, and mounted with non-aqueous medium (H-5700-60, Vector Laboratories). For the TMA staining evaluation, the staining intensity was graded as negative (0), weak (1), moderate (2), and strong (3). The H-Score (0–300) was calculated as (1 × % of weak staining) + (2 × % moderate staining) + (3 × % strong staining). Intensities of protein levels were independently evaluated by two well-trained investigators. The correlation between DDX3 and pDRP1 in the TMA was calculated.

### 2.15 Live-cell imaging

To visualize mitochondrial dynamics and utilization of FAO in 4T1-PT, 4T1-LM, and 4T1-LM shDDX3 cells after PA treatment, we performed holotomographic imaging on HT-X1 (Tomocube) to determine the lipid droplet deposition inside cells. Briefly, cells were seeded in a Tomodish provided by the manufacturer and allowed to reach 70 % confluency. The next day, cells were stained with MitoBright LT Red dye (MT11, Dojindo) and treated with PA. The dishes were placed in the Tomocube and imaged overnight according to the manufacturer's instructions. The obtained holotomographic images were processed and analyzed using equipment-assisted TomoAnalysis software. MitoBright LT Red staining was employed to ensure precision of mitochondria. The colocalization of lipid droplets and MitoBright LT Red dye was evaluated using a 3D holotomographic imaging analysis, in which lipid droplets were labeled in green.

### 2.16 Transmission electron microscopy (TEM)

Transmission electron microscopy (TEM) sample preparation was performed with assistance from the TMU Core Facility (Taipei, Taiwan). 4T1-PT, LM-shCon, and LM-shDDX3 cells were seeded in two-well chamber slides and treated with either palmitic acid (PA) or oleic acid (OA) overnight. Cells were then fixed in 0.1 M PHEM buffer containing 2 % paraformaldehyde and 0.2 % glutaraldehyde for 2 h at room temperature, followed by post-fixation with 1 % osmium tetroxide for 1.5 h. After dehydration through a graded ethanol series, samples were embedded in Epon resin and polymerized at 60 °C for 12 h. Ultrathin sections were stained with uranyl acetate and lead citrate, and examined using a Hitachi HT7700 transmission electron microscope at 100 kV and 1500 × magnification.

### 2.17 Bioinformatics

For analysis of gene expression levels across FA oxidation, glycolysis, glutaminolysis, and oxidative phosphorylation (OXPHOS) in breast cancer patients, the Molecular Taxonomy of Breast Cancer International Consortium (METABRIC) database was downloaded from the cBioPortal website (https://www.cbioportal.org/accessed on November 16, 2024) and analyzed. Protein expression levels in breast cancer patients derived from the Clinical Proteomic Tumor Analysis Consortium (CPTAC) dataset were downloaded from the cBioPortal website (accessed on November 16, 2024).

To analyze correlations of DDX3 with FA oxidation, glycolysis, glutaminolysis, and OXPHOS, the top 20 high DDX3-expressing breast cancer patients and top 20 low DDX3-expressing patients were selected. For analysis of gene expressions correlated with the FA oxidation signature, OXPHOS signature, glycolysis signature, and glutaminolysis signature, signature scores were calculated as follows. Every gene was transformed by Z-scores across all samples in The Cancer Genome Atlas (TCGA)_Breast Cancer (BRCA) or Cancer Cell Line Encyclopedia (CCLE) database. The list of these genes was summarized into a single value for each sample. Signature scores were subsequently used to calculate the Pearson correlation of all genes in the dataset.

### 2.18 Animal studies

All animal studies were performed based on guidelines and approval of the Animal Care and Use Committee of Taipei Medical University (LAC-2022-0311). All mice were maintained in a specific pathogen-free room on a 12:12-h light/dark cycle, and were fed autoclaved chow and water. Highly lung metastatic 4T1 cell lines were established as previously described [31]. To evaluate the inhibitory effect of DDX3-knockdown (KD) on tumorigenesis and metastasis, a syngeneic mouse model was conducted. Six-week-old female BALB/c mice were purchased from the National Laboratory Animal Center (Taipei, Taiwan) and randomized into three groups (*n* = 5 per group). In brief, 4T1-LM shCon and 4T1-LM shDDX3 cells (4 × 10^5^) were resuspended in a mixture of 100 μl of PBS and Matrigel, and orthotopically injected into the mammary fat pad of BALB/c mice. After 4 weeks when tumors in the shCon group had reached approximately 1000 mm^3^, the mice were sacrificed. Primary tumors, lungs, and livers were collected for further experiments. During the animal experiment, tumor formation and growth were assessed every 3 days. Tumor volume = Length × Width [2] × 0.52. Samples of tumors, lungs, and livers were stained with hematoxylin and eosin, and were further analyzed by light microscopy to evaluate the distribution of metastatic nodules in the second organs.

### 2.19 Statistical analyses

All data are expressed as the mean ± standard deviation (SD) of at least three independent experiments as indicated in the figure legends. Differences between two subgroups were determined by an unpaired, two-tailed Student's *t*-test unless stated otherwise. Statistical significance was indicated as follows: ∗*p* < 0.05, ∗∗*p* < 0.01, and ∗∗∗*p* < 0.001. Statistical analyses were carried out with GraphPad Prism 6.0 software.

## 3 Results

### 3.1 Metastatic breast tumor cells induce a metabolic shift towards FAO

**Fig. 1:**
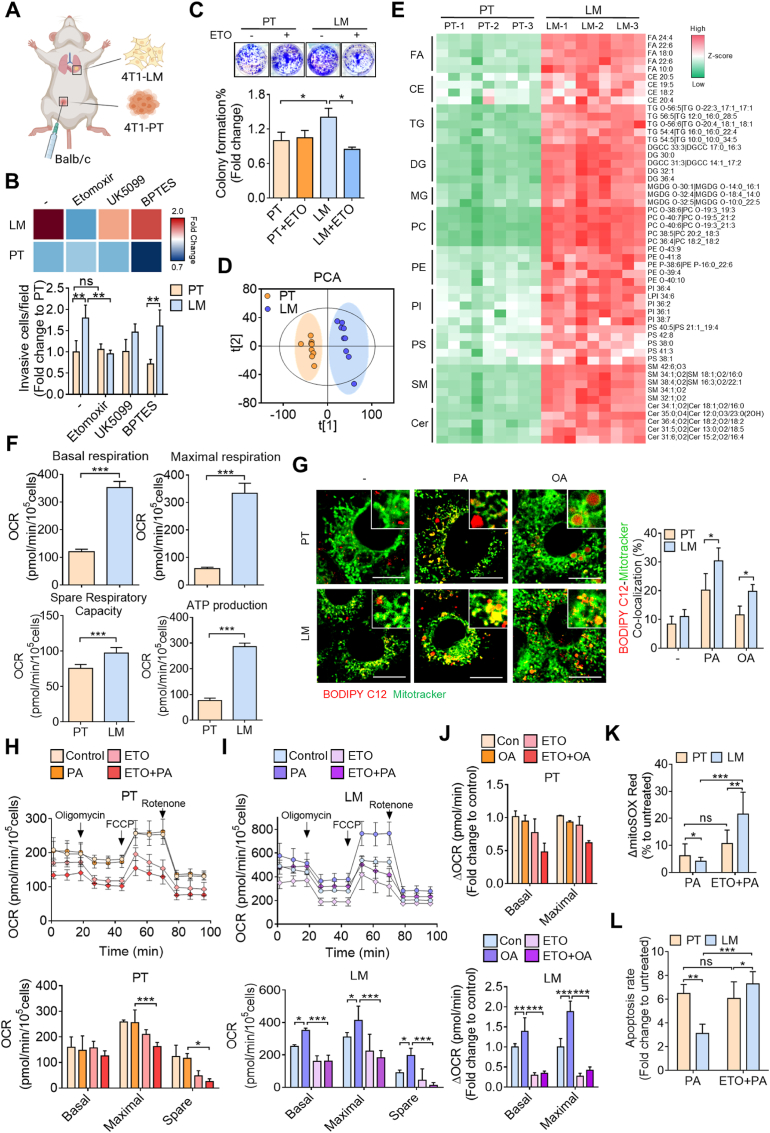
**Metastatic breast cancer cells induce a metabolic shift towards fatty acid oxidation (FAO).** (**A**) Schematic illustrating the establishment of 4T1-PT and 4T1-LM cells by *in vivo* selection. (**B**) Transwell invasion assay assessing the invasive capacities of 4T1-PT and 4T1-LM cells upon treatment with the indicated inhibitors. (**C**) Colony forming ability of 4T1-PT and 4T1-LM cells treated with or without Etomoxir (ETO). (**D**) Principal component analysis (PCA) showing lipidomics in 4T1-PT and 4T1-LM cells separated to two independent clusters. (**E**) Heatmap indicating lipidomic profiling in 4T1-PT and 4T1-LM cells. (**F**) Seahorse assay showing oxygen consumption rates (OCRs) in 4T1-PT and 4T1-LM cells. (**G**) Confocal images showing co-localization of BODIPY C12 and Mitotracker Green in PT/LM cells treated with or without palmitic acid (PA) or oleic acid (OA). Quantification colocalization analysis was performed. Scale bar, 10 μm. (**H–I**) Seahorse analysis showing OCRs of 4T1-PT (**H**) and 4T1-LM cells (**I**) treated with PA, ETO, or both. (**J**) OCRs of 4T1-PT and 4T1-LM cells treated with OA, ETO, or both. (**K, L**) Flow cytometry detecting changes in mitochondrial ROS levels (**K**) and apoptotic rates (**L**) in 4T1-PT and 4T1-LM cells induced by PA with or without ETO pretreatment. ∗*p* < 0.05, ∗∗*p* < 0.01, ∗∗∗*p* < 0.001 by an unpaired two-tailed *t*-test.

To investigate the metabolic vulnerability of metastatic breast cancer cells, we compared the metabolic dependency of lung metastatic (LM) and primary tumor (PT) cells isolated from an orthotopic breast cancer mouse model (Fig. 1A). Cells were treated with inhibitors targeting key metabolic pathways, including Etomoxir (ETO, an FAO inhibitor), BPTES (a glutaminolysis inhibitor), and UK5099 (a pyruvate dehydrogenase inhibitor). Notably, LM cells displayed higher migratory and invasive abilities than PT cells, as demonstrated by wound healing and transwell invasion assays (Fig. 1B–S1A). Among the tested inhibitors, ETO treatment most significantly impaired migration and invasion in LM cells, whereas PT cells were minimally affected. Consistently, colony formation assays showed that LM cells possessed greater clonogenic capacity, which was markedly reduced by ETO (Fig. 1C), suggesting a critical reliance on FAO to sustain metastatic potential. Together, these findings indicate that LM cells undergo a metabolic shift toward FAO dependency to support their aggressive behaviors. To substantiate this hypothesis, we performed a comparative lipidomic analysis between PT and LM cells. Lipidomic profiling revealed that over ten lipid subclasses were significantly enriched in LM cells, including several mitochondrial-associated phospholipids, such as phosphatidylcholine (PC), phosphatidylethanolamine (PE), phosphatidylinositol (PI), and phosphatidylserine (PS), suggesting enhanced lipid metabolism through FAO in metastatic breast cancer cells (Fig. 1D and E). Notably, ceramide (Cer) levels were intrinsically elevated in LM cells, indicating a metabolic adaptation to lipid-induced stress and further supporting the concept of FAO-driven metabolic reprogramming in these cells (Fig. 1D and E). Additionally, the Seahorse bioanalyzer revealed that LM cells predominantly utilized mitochondrial metabolism to meet energy demands compared to PT cells (Fig. 1F), highlighting the importance of mitochondrial metabolic pathways during metastasis.

To further clarify FAO utilization in metastatic breast tumor cells, LM and PT cells were treated with palmitic acid (PA) or oleic acid (OA). Confocal images showed that fatty acids displayed a pronounced colocalization with mitochondria in LM cells upon PA or OA treatment, while less colocalization was observed in PT cells (Fig. 1G). Seahorse analysis revealed that PA stimulation significantly increased OCRs—including basal, maximal, and spare respiratory capacities—in LM cells, whereas these enhancements were markedly suppressed by ETO treatment. In contrast, this effect was not observed in PT cells (Fig. 1H and I). A similar pattern was observed upon OA treatment (Fig. 1J), supporting the link between CPT-1-mediated FAO and metastatic potential. Metabolic stress can lead to excess mitochondrial ROS production and cellular damage. We found that PA treatment modestly increased mitochondrial ROS and induced less apoptosis in LM cells, whereas PT cells exhibited higher ROS levels and greater cell death. Notably, FAO inhibition under PA treatment caused a marked increase in mitochondrial ROS and apoptosis in LM cells compared to PT cells (Fig. 1K and L). These findings indicate that metastatic breast cancer cells have a greater capacity to cope with lipid-induced mitochondrial oxidative stress, yet are more vulnerable to FAO inhibition. As FAO enhances intracellular antioxidant capacity, this observation is consistent with our previous finding that LM cells are more resilient to oxidative stress [5], underscoring the essential role of FAO in supporting metastatic adaptation.

### 3.2 Metastatic breast tumor cells promote mitochondrial fission under FAO utilization

**Fig. 2:**
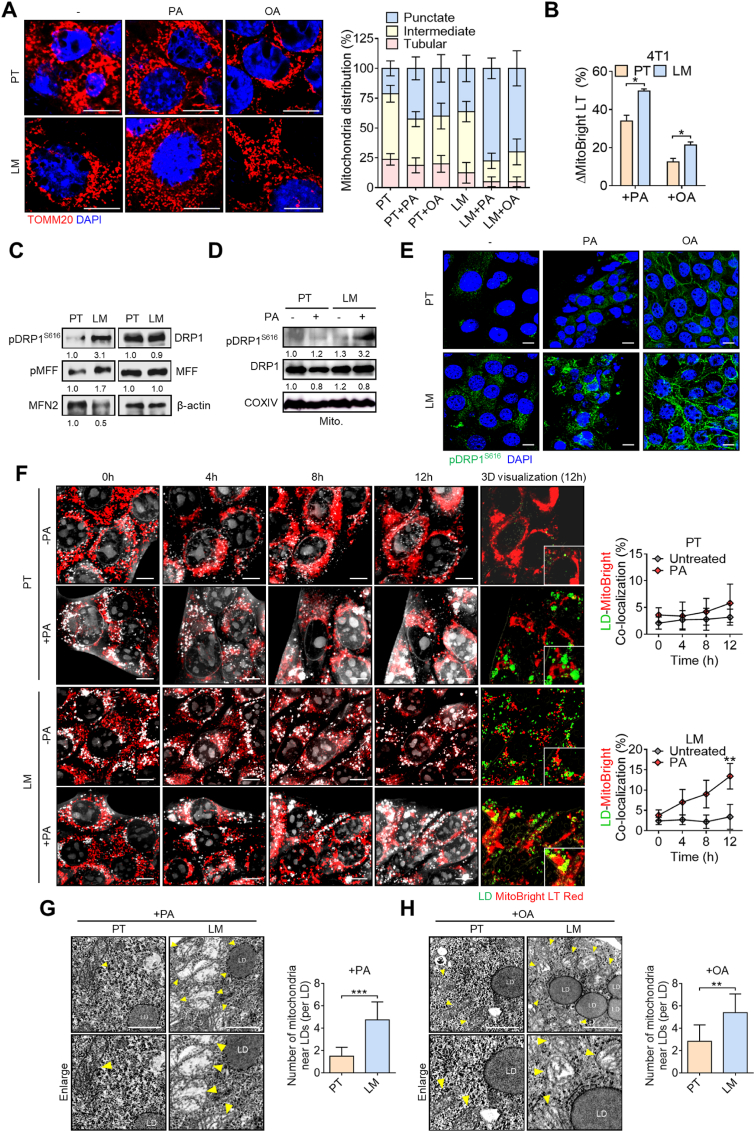
**Metastatic breast tumor cells promote mitochondrial fission under fatty acid oxidation (FAO).** (**A**) Confocal images showing the mitochondrial morphology of 4T1-PT and 4T1-LM cells treated with or without palmitic acid (PA) or oleic acid (OA). Distributions of punctate, intermediate, and tubular-like mitochondrial structures in the indicated conditions are shown. Scale bar, 10 μm. (**B**) Changes in the mitochondrial mass in primary tumor (PT) and metastatic (LM) cells induced by PA or OA as detected by a flow cytometric analysis. (**C**) Western blotting showing mitochondrial fission-associated regulators in 4T1-PT and 4T1-LM cells. (**D**) Western blot analysis indicating pDRP1^S616^ levels in PT/LM mitochondrial fractions induced with or without PA. (**E**) Confocal images of pDRP1^S616^ expression levels in 4T1-PT and 4T1-LM cells treated with or without PA or OA. Scale bar, 10 μm. (**F**) Live-cell images by holotomography showing mitochondrial morphology and colocalization of green-labeled lipid droplets (LD) and MitoBright Red-labeled mitochondria in PT/LM cells. Quantification of colocalization under the indicated conditions with the time course was analyzed. (**G-H**) TEM images showing more mitochondria near lipid droplets in LM cells compared to PT cells upon treatment with (**G**) PA or (**H**) OA. Scale bar, 1 μm. Quantification analysis of number of mitochondria near lipid droplets was performed. Scale bar, 10 μm ∗*p* < 0.05, ∗∗*p* < 0.01, ∗∗∗*p* < 0.001 by an unpaired two-tailed *t*-test. Representative immunoblot images are shown, with β-actin or COXIV serving as loading controls. Band intensities were quantified using ImageJ, normalized to the corresponding loading control, and presented as fold changes.

We further sought to investigate mitochondrial dynamics under FAO utilization in metastatic breast tumor cells. We found that the mitochondrial morphology of LM cells exhibited a more highly fragmented and punctate structure, compared to those in PT cells, which displayed greater distributions of intermediate and tubular structures (Fig. 2A). Strikingly, PA or OA treatment induced a more-fragmented mitochondrial morphology in LM cells, whereas it remained largely unchanged in PT cells (Fig. 2A). In parallel, the changes in mitochondrial mass were significantly increased after PA or OA treatment in LM cells, compared to those in PT cells (Fig. 2B), suggesting that PA induced a shift towards a fragmented mitochondrial phenotype in LM cells. Mitochondrial fission is mediated by several regulators, and we further found that DRP1 and MFF, mitochondrial fission regulators, did not increase in LM cells. However, phosphorylation of DRP1 at the S616 site, considered to activate DRP1 to drive mitochondrial fission, was drastically elevated in LM cells. Also, phosphorylation of MFF was increased in LM cells. On the contrary, the mitochondrial fusion factor, MFN2, declined in LM cells (Fig. 2C). Accordingly, results of the Western blot showed that PA remarkably induced DRP1^S616^ phosphorylation in mitochondrion fractions of LM cells, but not in PT cells (Fig. 2D). Confocal microscopic analyses revealed that both PA and OA significantly increased pDRP1^S616^ levels in LM cells compared to in PT cells (Fig. 2E), highlighting the specificity of pDRP1^S616^ induction in LM cells by fatty acids. Furthermore, live-cell images of holotomographic observations clearly showed that LM cells exhibited upregulated lipid droplets at a basal level (0 h), compared to those in PT cells. Nevertheless, these lipid droplets showed less localization in mitochondria. Notably, PA treatment gradually induced accumulation of lipid droplets in mitochondria only in LM cells. At the endpoint of PA induction, this co-localization was more greatly observed in LM cells than in PT cells (Fig. 2F), suggesting that mitochondrial fission is facilitated in metastatic breast cancer cells under FAO utilization. Furthermore, TEM analysis showed that mitochondria were more frequently positioned adjacent to lipid droplets upon PA or OA treatment in LM cells compared to PT cells, where such proximity was rarely observed. These findings indicate that both PA and OA stimulation promote stronger lipid–mitochondria interactions in LM cells (Fig. 2G and H). Collectively, this supports the idea that metastatic breast cancer cells enhance mitochondrial fission in response to FAO activation.

### 3.3 Elevation of DDX3 promotes FAO in association with mitochondrial fission in metastatic breast cancer cells

**Fig. 3:**
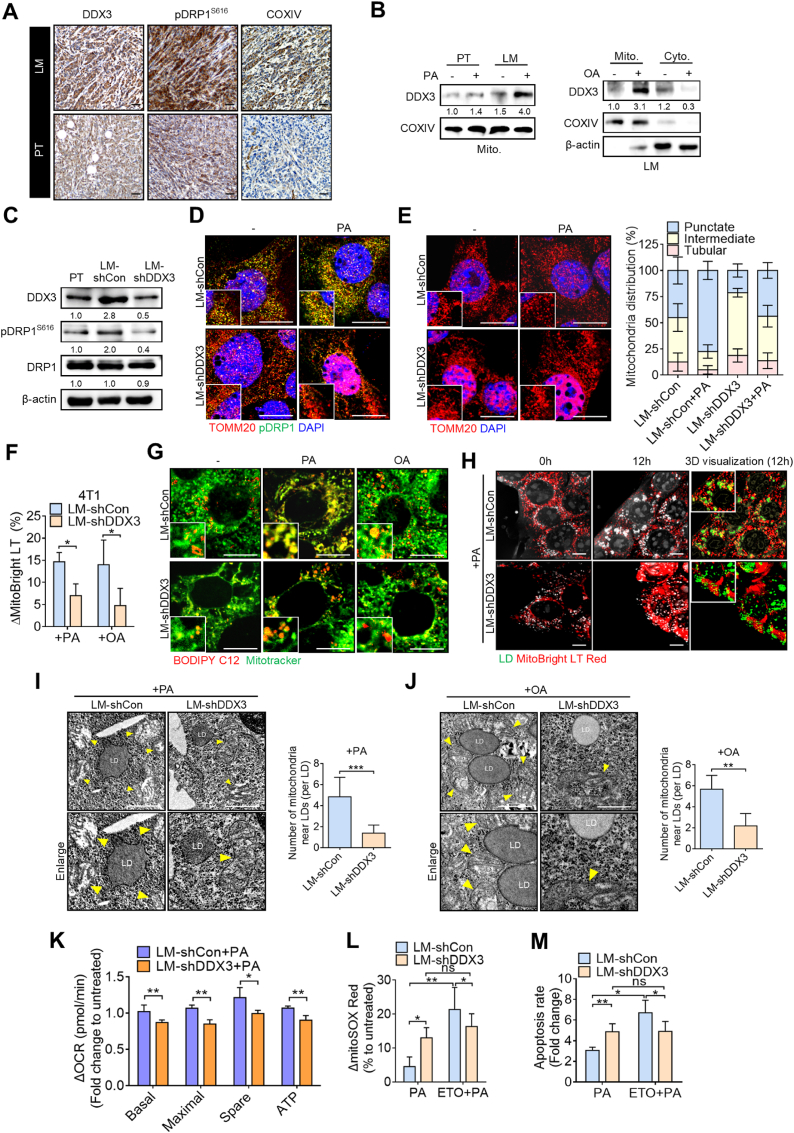
**Elevation of DDX3 promotes fatty acid oxidation (FAO) in association with mitochondrial fission in metastatic breast cancer cells.** (**A**) Representative IHC-stained images of DDX3-, pDRP1^S616^- and COXIV-positive staining per field of vision in tumor sections from LM cells (upper) and PT cells (lower). Scale bar, 30 μm. (**B**) DDX3 protein expression in mitochondrial and cytosolic fractions of PT and LM cells treated with or without palmitic acid (PA), and in LM cells treated with or without oleic acid (OA). COXIV, mitochondrial marker. (**C**) Western blotting showing DDX3 and pDRP1^S616^ levels in 4T1 whole-cell lysates. (**D, E**) Confocal images showing the pDRP1^S616^ and TOMM20 association (**D**) and mitochondrial morphology (**E**) in LM-shCon and LM-shDDX3 cells treated with or without PA. Distributions of mitochondrial structures under the indicated conditions are shown. Scale bar, 10 μm. (**F**) Alterations in mitochondrial mass in response to PA or OA treatment in LM-shCon and LM-shDDX3 cells, as determined by flow cytometric analysis. (**G**) Confocal images indicating colocalization of BODIPY C12 and Mitotracker Green in LM-shCon and LM-shDDX3 cells treated with or without PA or OA. Scale bar, 10 μm. (**H**) Live-cell images by holotomography showing the mitochondrial morphology and colocalization of green-labeled LD and MitoBright Red-labeled mitochondria in LM-shCon and LM-shDDX3 cells. Scale bar, 10 μm. (**I-J**) TEM images showing mitochondria-lipid droplet association in LM-shCon and LM-shDDX3 cells upon PA (**I**) or OA (**J**) treatment. Scale bar, 1 μm. (**K**) Seahorse analysis demonstrating changes in oxygen consumption rates (OCRs) in LM-shCon and LM-shDDX3 cells which were both treated with PA. (**L, M**) Flow cytometry detecting changes of mitochondrial ROS levels (**L**) and apoptotic rates (**M**) in LM-shCon and LM-shDDX3 cells induced by PA with or without Etomoxir pretreatment. ∗*p* < 0.05, ∗∗*p* < 0.01, ∗∗∗*p* < 0.001 by an unpaired two-tailed *t*-test. Representative immunoblot images are shown, with β-actin or COXIV serving as loading controls. Band intensities were quantified using ImageJ, normalized to the corresponding loading control, and presented as fold changes.

DDX3 has been reported to be upregulated in triple-negative and metastatic breast cancers and linked to poor clinical outcomes [[35], [36], [37]]. Our previous study demonstrated that DDX3 expression is significantly elevated in metastatic breast cancer lesions compared to primary tumors, and functionally contributes to cancer stemness and chemoresistance [31]. We thus examined the involvement of DDX3 in mitochondrial fission and FAO. The IHC analysis revealed that DDX3 expression along with pDRP1^S616^ and the mitochondrion marker, COXIV, were upregulated in metastatic tumor tissues (Fig. 3A). To further investigate how DDX3 regulates mitochondrial fission, we purified mitochondrial and cytosolic fractions and found that PA stimulation markedly increased mitochondrial DDX3 levels in LM cells, whereas its mitochondrial localization remained unchanged in PT cells (Fig. 3B). Similarly, OA treatment also induced mitochondrial translocation of DDX3 in LM cells, suggesting that this response is specific to metastatic cells (Fig. 3B). Therefore, we wondered if DDX3 regulates DRP1^S616^ phosphorylation. DDX3 was stably knocked-down in LM cells, which expressed higher DDX3 protein levels as well as pDRP1^S616^. Results showed that DDX3 silencing reduced DRP1^S616^ phosphorylation without affecting the total DRP1 protein level (Fig. 3C). Simultaneously, confocal microscopy showed that pDRP1^S616^ expression levels in mitochondria were higher in LM-shCon cells at the basal level, and the PA-induced increase in DRP1^S616^ phosphorylation was abrogated in LM-shDDX3 cells (Fig. 3D). Consistently, DDX3 knockdown promoted a tubular mitochondrial morphology in LM cells (Fig. 3E). PA-induced mitochondrial fragmentation was abolished in DDX3-depleted cells compared to LM-shCon cells (Fig. 3E). In addition, the changes in mitochondrial mass triggered by PA or OA were more pronounced in LM-shCon cells, while these responses were attenuated in LM-shDDX3 cells (Fig. 3F). Confocal images demonstrated that the colocalization between fatty acids and mitochondria was markedly enhanced by PA or OA in control cells, but significantly reduced in DDX3-knockdown cells (Fig. 3G). Similarly, live-cell holotomography imaging revealed increased lipid droplet–mitochondria colocalization in LM-shCon cells following PA treatment, which was impaired in LM-shDDX3 cells (Fig. 3H). Consistent with this, TEM analysis showed that PA or OA stimulation led to abundant mitochondria adjacent to lipid droplets in LM-shCon cells, whereas LM-shDDX3 cells exhibited a notable reduction in mitochondria near lipid droplets, indicating that DDX3 is required for maintaining lipid–mitochondria interactions (Fig. 3I and J). These data collectively suggest that DDX3 promotes pDRP1-mediated mitochondrial fission and facilitates FAO during metastatic progression. The Seahorse analysis also revealed that PA treatment induced much greater changes in basal respiration, maximal respiration, ATP production, and spare respiratory capacity in LM-shCon cells, compared to LM-shDDX3 cells (Fig. 3K), demonstrating that DDX3 promotes mitochondrial fission and facilitates FAO utilization. We next investigated whether high DDX3 expression rendered metastatic cells particularly vulnerable to FAO inhibition. Results showed that DDX3-KD increased mitochondrial ROS levels by about 2-fold in the presence of PA (Fig. 3L), which was accompanied by induction of cell apoptosis (Fig. 3M), corroborating that DDX3 expression facilitated FAO and reduced mitochondrial oxidative stress. Accordingly, inhibition of FAO in LM cells sharply increased mitochondrial ROS production and cell death by 4-fold, while it showed a modest effect on LM/shDDX3 cells (Fig. 3L and M). These data indicated that DDX3 facilitates FAO and alleviates mitochondrial oxidative stress.

### 3.4 DDX3 facilitates FAO in association with mitochondrial fission in TNBC cells

**Fig. 4:**
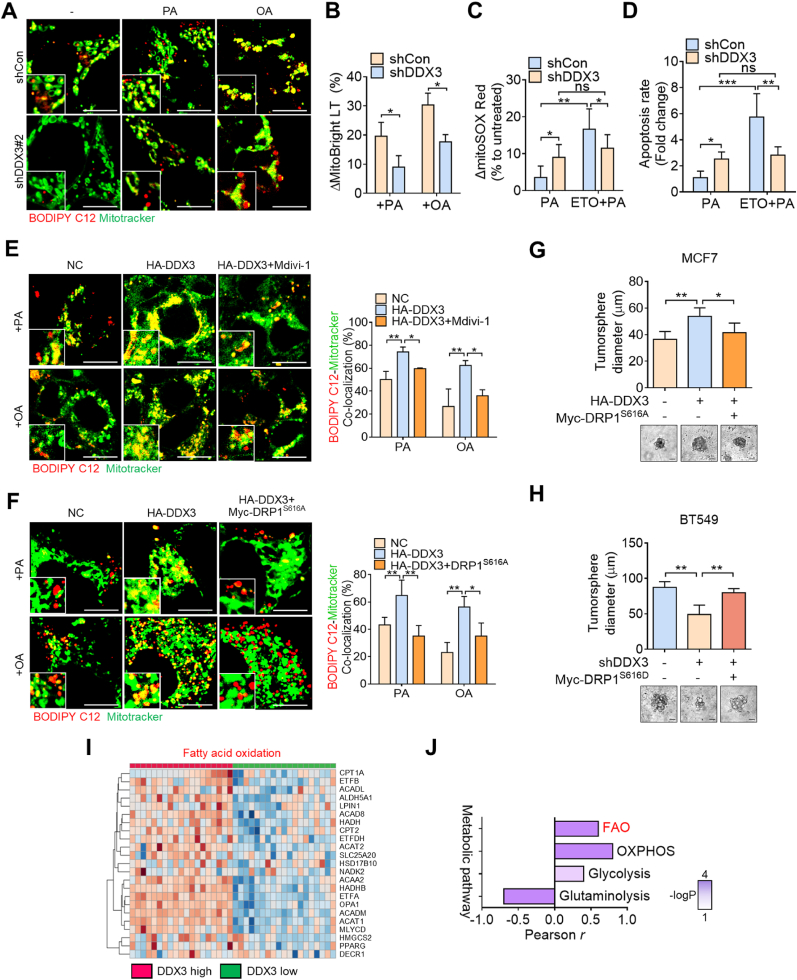
**DDX3 facilitates FAO in association with mitochondrial fission in TNBC cells.** (**A**) Confocal images indicating colocalization of BODIPY C12 and Mitotracker Green in BT549 shCon and shDDX3 cells treated with or without palmitic acid (PA) or oleic acid (OA). Scale bar, 10 μm. (**B**) Flow cytometry evaluating changes in the mitochondrial mass of BT549 shCon and shDDX3 cells induced by PA or OA. (**C, D**) Flow cytometry detecting changes of mitochondrial ROS levels (**C**) and apoptotic rates (**D**) in BT549 shCon and shDDX3 cells. (**E**) Confocal images showing the colocalization of fatty acids and mitochondria in control and DDX3-overexpressing MCF7 cells, with or without Mdivi-1 pretreatment, upon stimulation with PA or OA. Scale bar, 10 μm. (**F**) Confocal images showing the colocalization of fatty acids and mitochondria in control and DDX3-overexpressing MCF7 cells co-transfected with or without the DRP1 S616A mutant, followed by PA or OA treatment. Scale bar, 10 μm. (**G**) Tumorsphere formation assay of MCF7 cells transfected with pcDNA control, DDX3 overexpression, or co-overexpression of DDX3 and DRP1^S616A^. Scale bar, 20 μm. (**H**) Tumorsphere formation assay of BT549 cells with shCon, shDDX3, or shDDX3 rescued with DRP1^S616D^ overexpression. Scale bar, 20 μm. (**I**) Heatmap showing the correlation between DDX3 and FAO-related gene expressions in breast cancer patients by analyzing the METABRIC database. (**J**) Correlations between DDX3 and metabolic pathways as indicated in breast cancer patients, which was derived from the analysis in (**I**). ∗*p* < 0.05, ∗∗*p* < 0.01, ∗∗∗*p* < 0.001 by an unpaired two-tailed *t*-test.

We further validated that this phenotype could be recapitulated in human triple-negative breast cancer (TNBC) cells, in which we previously identified elevated DDX3 expression [31]. DDX3 was stably knocked-down in BT549 cells which expresses a higher level of DDX3. Consistent with the results described above, DDX3 knockdown in BT549 cells reduced the colocalization between fatty acids and mitochondria (Fig. 4A), and attenuated mitochondrial mass changes in response to PA or OA stimulation (Fig. 4B). Similarly, inhibition of FAO aggravated mitochondrial ROS levels and cell death in DDX3-expressing cells, while the silencing of DDX3 showed opposite effects (Fig. 4C and D). To further validate the role of DDX3-mediated mitochondrial fission in facilitating FAO, we assessed the effect of Mdivi-1, a mitochondrial fission inhibitor, in DDX3-overexpressing cells. Confocal imaging revealed that DDX3 overexpression enhanced FAO upon PA or OA treatment, while this effect was significantly diminished by Mdivi-1 (Fig. 4E). Because Mdivi-1 was also reported to reversibly inhibit mitochondrial complex I [38], we further validated the role of DRP1 by introducing a phosphorylation-deficient DRP1 S616A mutant to clarify its contribution to DDX3-driven FAO. Thus, a phosphorylation-deficient DRP1 S616A mutant plasmid was transfected into DDX3-overexpressing cells, and a phosphomimetic DRP1 S616D mutant was transduced into DDX3-depleted cells. Notably, DDX3 overexpression increased the colocalization of fatty acids and mitochondria upon lipid stimulation, whereas this effect was abolished by DRP1 S616A expression (Fig. 4F). Functionally, DDX3 overexpression significantly promoted tumorsphere formation, while this effect was reversed by co-expression of DRP1 S616A (Fig. 4G), indicating that DRP1 phosphorylation is essential for DDX3-driven metabolic rewiring and stem-like properties. Consistently, in BT549 cells, DDX3 knockdown impaired tumorsphere formation, while re-expression with DRP1^S616D^ restored the sphere-forming capacity. A similar pattern was observed in cell proliferation (Fig. 4H–S2A). These results collectively suggest that DDX3 promotes fatty acid utilization and stemness through DRP1 phosphorylation at serine 616. Moreover, analysis of the TCGA breast cancer dataset showed that DDX3 expression positively correlated with FAO and OXPHOS gene signatures, but not with glycolysis, and was inversely correlated with glutaminolysis (Fig. 4I, J, S3A). Together, these findings demonstrate that DDX3 drives a metabolic shift toward FAO in metastatic breast cancer cells by promoting mitochondrial fission and maintaining mitochondrial function under lipid-rich conditions, supporting the notion that DDX3 contributes to metabolic reliance on FAO in metastatic contexts.

### 3.5 DDX3 interacts with DRP1 and facilitates mitochondrial fission

**Fig. 5:**
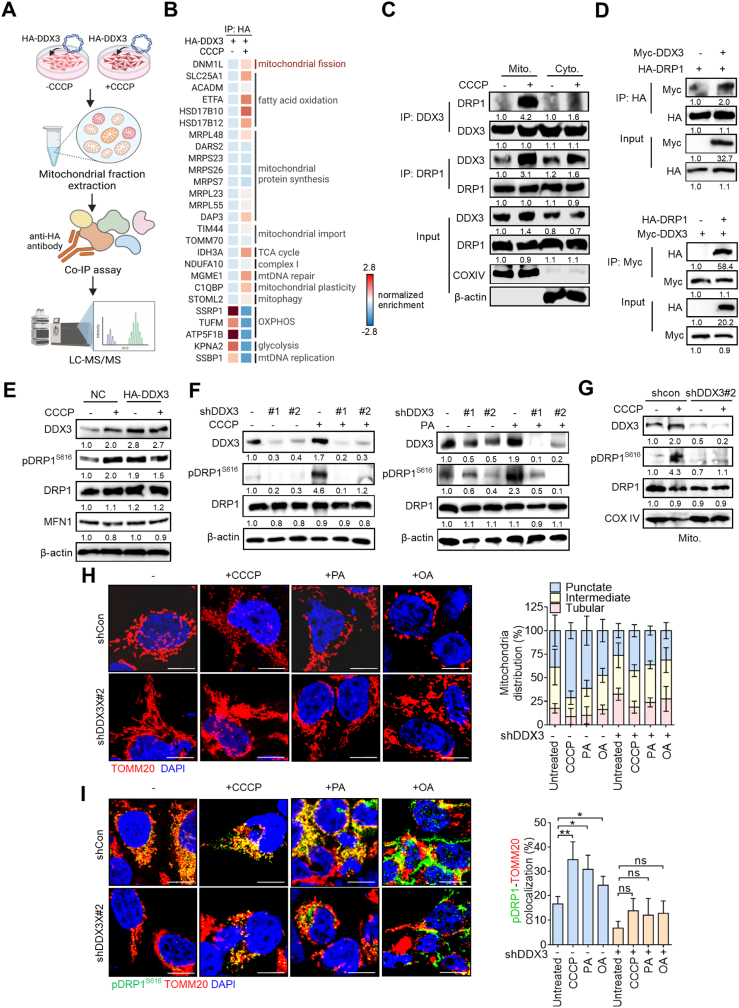
**DDX3 interacts with DRP1 and facilitates mitochondrial fission.** (**A**) Schematic diagram describing the progression, which identified DDX3-interacting mitochondrial proteins upon CCCP induction. (**B**) Heatmap indicating DDX3-interacitng proteins in mitochondria upon CCCP treatment identified by LC-MS/MS. (**C**) Co-IP assay showing the interaction between DDX3 and DRP1 upon CCCP induction in mitochondrial and cytosol fractions. (**D**) Co-IP assay showing the interaction between exogenous DDX3 and DRP1. (**E**) Western blotting showing pDRP1^S616^ levels in DDX3-overexpressing MCF7 cells stimulated with or without CCCP. (**F**) Western blotting indicating pDRP1^S616^ levels in DDX3-silenced BT549 cells treated with or without CCCP or PA. (**G**) Expression levels of pDRP1^S616^ in mitochondrial fractions of BT549 shCon and shDDX3 cells treated with or without CCCP. (**H, I**) Confocal images visualizing mitochondrial morphology (**H**) and the colocalization of pDRP1^S616^ and TOMM20 under the indicated conditions (**I**) in BT549 shCon and shDDX3 cells treated with or without CCCP, PA or OA. Distributions of mitochondrial structures under the indicated conditions are shown. Scale bar, 10 μm. ∗*p* < 0.05, ∗∗*p* < 0.01 by an unpaired two-tailed *t*-test. Representative immunoblot images are shown, with β-actin or COXIV serving as loading controls. Band intensities were quantified using ImageJ, normalized to the corresponding loading control, and presented as fold changes. For IP analysis, quantification was normalized to the first lane.

To further identify DDX3's interactome in regulating mitochondrial fission, cells were overexpressed with HA-tagged DDX3 and treated with 10 μM of CCCP for 6 h, which can induce mitochondrial fission with limited cytotoxic effects. Mitochondrial fractions were respectively extracted and immunoprecipitated with an anti-HA antibody and subjected to an LC-MS/MS analysis (Fig. 5A). Results showed that CCCP treatment obviously induced an association of DDX3 with several mitochondrial-associated proteins. Of interest, we found that DRP1 (encoded by *DNM1L*) was enriched with DDX3 after CCCP treatment (Fig. 5B), indicating that DDX3 might facilitate mitochondrial fission through interacting with DRP1. A Co-IP assay further confirmed the endogenous association between DDX3 and DRP1 upon CCCP induction, especially in mitochondrial fractions (Fig. 5C). Exogenous expressions of DDX3 and DRP1 also validated the interaction with each other (Fig. 5D).

We further examined the role of DDX3 in regulating mitochondrial fission under stress conditions. In mock cells, CCCP treatment elevated both DDX3 and pDRP1^S616^ levels. Interestingly, DDX3 overexpression alone was sufficient to increase pDRP1^S616^ levels even in the absence of CCCP, while MFN1 expression remained unchanged. Moreover, CCCP treatment did not further enhance pDRP1^S616^ levels in DDX3-overexpressing cells, suggesting that DDX3 may function upstream of stress-induced DRP1 activation (Fig. 5E). On the contrary, CCCP or PA treatment did not upregulate DRP1^S616^ phosphorylation in DDX3-silenced cells in either total cell lysates or mitochondria fractions (Fig. 5F and G). These data were in line with the above findings, indicating that DDX3 overexpression obligatorily promotes mitochondrial fission. Confocal images also demonstrated that the mitochondrial morphology was significantly more punctate and fragmented upon CCCP, PA or OA induction compared to untreated cells, while the mitochondrial structure in DDX3-KD cells exhibited more tubular-like structures (Fig. 5H). We also found that CCCP, PA or OA treatment induced pDRP1 colocalization with TOMM20 in mock cells, while it was abrogated in DDX3-KD cells (Fig. 5I). In support of these findings, treatment with the DDX3 inhibitor RK-33 similarly reduced the colocalization between mitochondria and fatty acids, and impaired pDRP1^S616^ protein levels and the recruitment of pDRP1 to mitochondria. These observations are consistent with the effects of genetic DDX3 knockdown (Fig. S4A–S4C). These findings suggest that DDX3 translocates to mitochondria where it regulates DRP1 phosphorylation and thereby facilitates mitochondrial fission and FAO utilization.

### 3.6 DDX3 regulates FAO via its association with DRP1 and CDK1

**Fig. 6:**
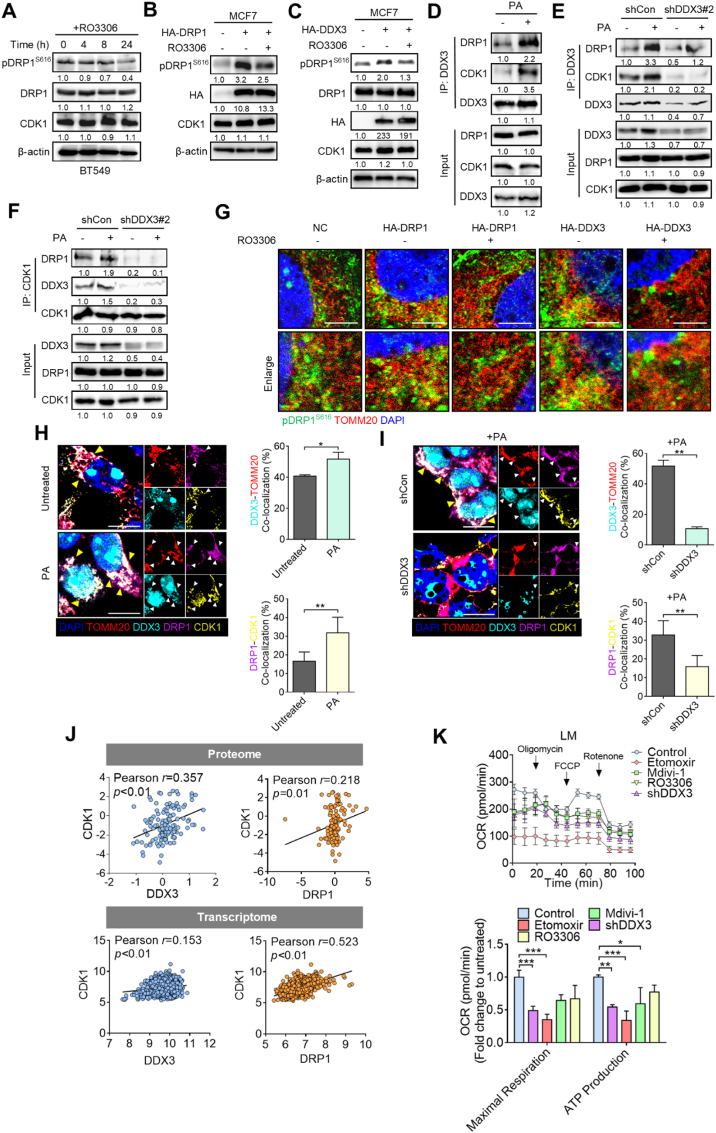
**DDX3 promotes fatty acid oxidation (FAO) via its association with DRP1 and CDK1.** (**A**) Western blotting assay showing pDRP1^S616^ levels in RO3306-treated BT549 cells. (**B**) Protein levels of pDRP1^S616^ in DRP1-overexpressing MCF7 cells treated with or without RO3306. (**C**) Protein levels of pDRP1^S616^ levels in DDX3-overexpressing MCF7 cells treated with or without RO3306. (**D**) Protein levels of DRP1 and CDK1 in palmitic acid (PA)-treated BT549 cells co-immunoprecipitated with DDX3 and analyzed by Western blotting. (**E**) Western blot analysis of endogenous DRP1 and CDK1 co-immunoprecipitated with DDX3 in PA-treated BT549 shCon or shDDX3 cells. (**F**) Western blot analysis of endogenous DRP1 and DDX3 co-immunoprecipitated with CDK1 in PA-treated BT549 shCon or shDDX3 cells. (**G**) Confocal images indicating colocalization of pDRP1^S616^ and TOMM20 in DRP1- or DDX3-overexpressing MCF7 cells. Scale bar, 5 μm. (**H**) Multiplex IF staining showing increased colocalization of DDX3, DRP1, CDK1, and TOMM20 in PA-treated BT549 cells. Yellow arrow indicates mitochondrial DDX3–CDK1–DRP1 complex. (**I**) Multiplex IF staining showing reduced CDK1-DRP1 colocalization and disrupted mitochondrial localization of the complex (green arrow) in PA-treated shDDX3 BT549 cells. Quantifications of DDX3–TOMM20 colocalization and DRP1–CDK1 colocalization are shown. Scale bar, 10 μm. (**J**) Database analysis of the correlation of DDX3/CDK1 with DRP1/CDK1 in proteome and transcriptome levels respectively derived from CPTAC and TCGA database. (**K**) Seahorse assay in 4T1-LM cells treated with Etomoxir, Mdivi-1, RO3306, or DDX3-knockdown. ∗*p* < 0.05, ∗∗*p* < 0.01, ∗∗∗*p* < 0.001 by an unpaired two-tailed *t*-test. Representative immunoblot images are shown, with β-actin or COXIV serving as loading controls. Band intensities were quantified using ImageJ, normalized to the corresponding loading control, and presented as fold changes. For IP analysis, quantification was normalized to the first lane.

Given that DDX3 interacts with and promotes phosphorylation of DRP1, and suppression of DDX3 did not affect the DRP1 protein level, we further excavated the molecular regulation underlying DDX3-mediated mitochondrial fission, by examining the involvement of CDK1, a known regulator of DRP1^S616^ phosphorylation [39,40]. Cells were treated with the CDK1 inhibitor RO3306. Time-course analysis revealed that RO3306 reduced DRP1 S616 phosphorylation without altering total DRP1 levels (Fig. 6A), and this inhibitory effect was also observed in DRP1-overexpressing cells (Fig. 6B), supporting CDK1-dependent regulation of DRP1 phosphorylation. Importantly, DDX3-induced DRP1 S616 phosphorylation was similarly attenuated by RO3306 treatment, while total DRP1 expression remained unaffected (Fig. 6C). A co-IP assay showed that PA or CCCP stimulation increased the interaction of DDX3 with both DRP1 and CDK1 (Fig. 6D–S5A). However, this association was markedly impaired in DDX3-knockdown cells under the same condition (Fig. 6E). Consistently, depletion of DDX3 significantly reduced the CDK1-DRP1 interaction regardless of PA treatment (Fig. 6F), and a similar disruption was observed upon treatment with the DDX3 inhibitor RK33 (Fig. S5B). Confocal analysis further demonstrated that overexpression of either DDX3 or DRP1 enhanced mitochondrial localization of pDRP1^S616^, which was abolished by CDK1 inhibition (Fig. 6G), reinforcing the requirement of CDK1 for DDX3-driven DRP1 activation. Multiplex IF staining revealed that PA stimulation promoted the colocalization of DDX3, DRP1, CDK1, and the mitochondrial marker TOMM20, suggesting the formation of a DDX3–CDK1–DRP1 complex at mitochondria (Fig. 6H). In contrast, DDX3 silencing disrupted the mitochondrial colocalization of these proteins under the same treatment (Fig. 6I). Quantification further confirmed that PA increased DDX3–TOMM20 colocalization, consistent with DDX3 translocation to mitochondria upon metabolic stress, while CDK1–DRP1 colocalization was diminished in the absence of DDX3 (Fig. 6H and I). In line with these findings, analysis of clinical breast cancer datasets revealed that CDK1 expression positively correlated with both DDX3 and DRP1 at the transcriptomic and proteomic levels (Fig. 6J). Functionally, suppression of DDX3 impaired OXPHOS activity to a similar extent as ETO, Mdivi-1, or RO3306 treatment (Fig. 6K), underscoring the essential role of the DDX3–CDK1–DRP1 axis in supporting mitochondrial bioenergetic capacity.

### 3.7 DDX3 and DRP1 facilitate breast cancer tumorigenesis and metastasis

**Fig. 7:**
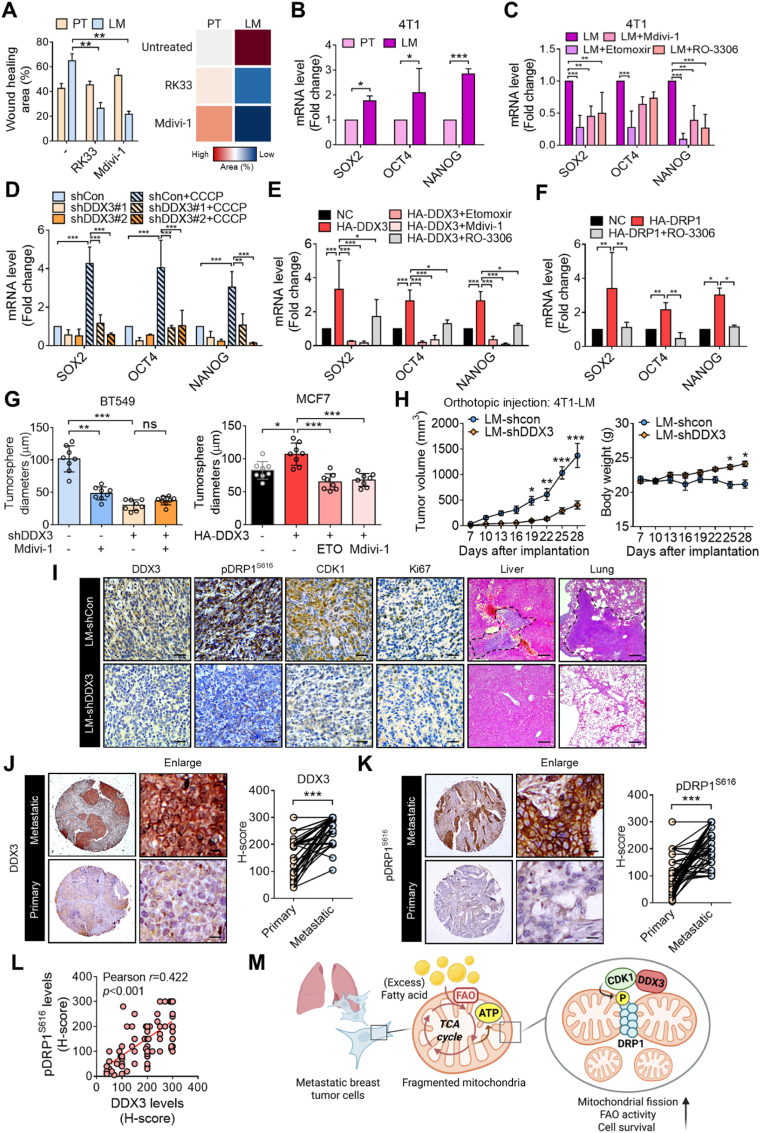
**Upregulation of the DDX3-DRP1-CDK1 axis facilitates breast cancer tumorigenesis and metastasis.** (**A**) Wound-healing assay evaluating the migratory ability of 4T1-PT and 4T1-LM cells treated with the indicated inhibitors. (**B**) Stemness-associated gene expression levels detected by an RT-qPCR assay. (**C**) RT-qPCR analysis evaluating the inhibitory effects of the indicated inhibitors on stemness-related gene expression levels in metastatic (LM) cells. (**D**) Stemness-associated gene mRNA levels in BT549 cells. (**E**, **F**) RT-qPCR analysis evaluating the inhibitory effects of the indicated inhibitors on stemness-related gene expression levels in DDX3-overexpressing (**E**) and DRP1-overexpressing (**F**) MCF7 cells. (**G**) Sphere-formation assay in BT549 and MCF7 cells. (**H**) 4T1-LM-shCon and 4T1-LM-shDDX3 cells were orthotopically injected into Balb/c mice. Tumor sizes (left) were monitored and analyzed (*n* = 6 mice per group). Body weights were estimated (right). (**I**) Representative IHC-stained images of DDX3-, pDRP1^S616^-, CDK1-, and Ki67-positive staining per field of vision in tumor sections (left panel) from primary tumors. H&E staining of liver and lung sections (right panel). Metastatic tumors are cropped. Scale bar, 50 μm. (**J**, **K**), Representative IHC-stained images of DDX3-and pDRP1^S616^-positive staining per field of vision in breast cancer patient tumor samples from a paired tissue microarray (TMA). Scale bar, 20 μm. Quantitation was analyzed based on H-scores. Fifty pairs were counted. (**L**) Correlation between expression levels of DDX3 and pDRP1^S616^ according to H-scores. (**M**) Graphic abstract describing the findings in this study. ∗*p* < 0.05, ∗∗*p* < 0.01, ∗∗∗*p* < 0.001 by an unpaired two-tailed *t*-test.

To validate the malignant role of DDX3-high LM cells *in vitro*, the DDX3 chemical inhibitor, RK33, was added to PT and LM cells. Indeed, the migratory ability of LM cells was effectively inhibited by RK33 treatment, emphasizing the critical role of DDX3 in LM aggressiveness (Fig. 7A). The migratory ability of LM cells was suppressed by Mdivi-1 treatment as well, supporting that mitochondrial fission is associated with the aggressiveness of LM cells (Fig. 7A). LM cells displayed higher stemness features than PT cells, which was evaluated by SOX2, OCT4, and NANOG mRNA levels (Fig. 7B). Notably, stemness-associated gene expression levels generally decreased with Mdivi-1, Etomoxir, and RO3306 treatment, indicating that mitochondrial fission, FAO utilization, and CDK1 activity significantly promote stemness in LM cells (Fig. 7C). Similarly, CCCP, a mitochondrial fission inducer, greatly increased stemness-related gene expression levels in BT549 shCon cells, while CCCP was unable to recover these gene levels, which were reduced by DDX3-KD, suggesting that DDX3 regulates mitochondrial fission to promote stemness in breast cancer cells (Fig. 7D). DDX3 overexpression enhanced stemness-related gene expression levels, while their inductions were respectively decreased by Etomoxir, Mdivi-1, and RO3306 (Fig. 7E), indicating that DDX3 mediates FAO, mitochondrial fission, and CDK1 recruitment to promote stemness. DRP1 overexpression induced gene-associated expression levels, while these enhancements were reduced by RO3306 (Fig. 7F), indicating that the DRP1-CDK1 axis contributes to stemness. Similar patterns were clearly observed in the tumorsphere formation assay (Fig. 7G).

To further validate the tumorigenic and metastatic capabilities of DDX3 *in vivo*, an orthotopic breast cancer metastasis model in syngeneic mice was conducted. Obviously, suppression of DDX3 in LM cells significantly inhibited primary tumor growth (Fig. 7H). Body weights of LM-shCon tumor-bearing mice were significantly lower than those of LM-shDDX3 tumor-bearing mice, which still gradually gained weight (Fig. 7H). Histological examinations demonstrated that DDX3-KD remarkably suppressed lung and liver metastases (Fig. 7I). IHC staining also indicated that DDX3 suppression effectively inhibited Ki67 expression and downregulated pDRP1^S616^ and CDK1 (Fig. 7I). Clinically, IHC analyses of the TMA showed that DDX3 and DRP1 expression levels were both upregulated in lymph nodes with metastatic tumors compared to matched primary breast tumors (Fig. 7J and K). Expression levels of pDRP1^S616^ were positively correlated with DDX3 levels (Fig. 7L). Our findings described the DDX3-DRP1-CDK1 axis mediating mitochondrial fission upon FAO utilization in metastatic breast cancer cells. Mechanistically, upon stimulation with excess lipids, DDX3 translocates to mitochondria and associates with DRP1 and CDK1. CDK1 is recruited to phosphorylate DRP1 S616 that promotes mitochondrial fission and fragmentation, thus facilitating stemness characteristics and metastasis of breast cancer cells (Fig. 7M).

## 4 Discussion

Mitochondrial homeostasis plays an indispensable role in tumor progression. We recently identified that DDX3 promotes mitochondrial biogenesis and mitophagy in breast cancer cells [31]. In line with our findings, DDX3 was observed to maintain mitochondrial protein quality control in *Leishmania* [41], and ablation of DDX3 by shRNA-KD or by chemical inhibitor treatment decreased proteins involved in mitochondrial translation and OXPHOS [42], highlighting the importance of DDX3 in mitochondrial homeostasis. So far, the oncogenic role of DDX3 appears dominant due to its RNA helicase activity, which enhances mRNA stability and protein translation [[43], [44], [45], [46], [47]]. We previously reported that DDX3 promotes mitophagy through its binding with PINK1 mRNA thereby facilitating PINK1 translation [31]. However, we also found that DDX3 is translocated to mitochondria, and its expression is associated with mitochondrial dynamics. In the present study, we further uncovered for the first time that DDX3 modulates mitochondrial dynamics through protein interactions with DRP1 and CDK1, which further enhances DRP1 phosphorylation. Overexpression of DDX3 boosted the pDRP1^S616^ level, while the total DRP1 protein expression remained unchanged. The increase in pDRP1^S616^ protein levels induced by DDX3 overexpression was impeded by CDK1 inhibition (Fig. 6). Unlike major functions of DDX3 previously found in other studies, these findings suggested that DDX3 might function as a chaperone in regulating mitochondrial dynamics, in which DDX3 interacts with both DRP1 and CDK1 to promote CDK1-mediated DRP1 phosphorylation at the S616 site. Interestingly, one study demonstrated that DDX3 was identified by MS to be associated with mitochondrial DDX28 and DHX30 interactome [42], which was shown to be responsible for the assembly of mitochondrial ribosomes [48,49]. These proteins were downregulated upon RK-33 treatment or DDX3 depletion, indicating that DDX3's helicase activity might be involved in mitochondrial ribosome assembly to further facilitate the mitochondrial bioenergetic machinery and efficiency. This is in line with our IP-MS data that DDX3 increased its association with proteins responsible for mitochondrial translation including MRPL23, MRPL55, MRPS23, and MRPS26 (Fig. 5). Together, these data underscore the importance of DDX3 in mitochondrial dynamics and biogenesis.

Mitochondrial fission is shown to promote tumor aggressiveness. In our present study, we identified that increased DRP1 S616 phosphorylation was concomitant with MFN2 downregulation in LM cells. However, our IP-MS analysis identified that DRP1 was the only mitochondrial fission-related protein interacting with DDX3, and this association was further enhanced by CCCP stimulation. Notably, mitochondrial fusion factors such as MFN2 and OPA1 were not detected in the DDX3-interacting proteome, suggesting a degree of selectivity toward the fission machinery (Fig. 5). Consistent with this, DDX3 overexpression increased DRP1 phosphorylation at serine 616 and promoted mitochondrial fragmentation, while DDX3 knockdown or pharmacological inhibition resulted in elongated mitochondrial morphology and reduced FAO activity (Fig. 5). Although MFN2 expression was found to be decreased in LM cells compared to PT cells (Fig. 2), the absence of physical interaction between DDX3 and MFN2, along with the consistent activation of DRP1, supports the interpretation that DDX3 primarily regulates mitochondrial fission through the DRP1 axis. Of note, previous studies have shown that excessive mitochondrial fission can suppress MFN2 expression or stability, either as a compensatory response or through degradation during mitophagy, especially under metabolic stress conditions [50]. Thus, it is possible that the elevated DRP1 activity in LM cells contributes to the downregulation of MFN2 as a secondary consequence [51]. Additionally, while metastatic breast cancer cells are generally biased toward a fragmented mitochondrial morphology, such remodeling likely involves multiple layers of regulation beyond DDX3 alone. Nevertheless, our findings highlight DDX3 as a key upstream effector that promotes mitochondrial fission by modulating DRP1 activation and recruitment to mitochondria. These findings collectively underscore the specificity and functional importance of the DDX3-DRP1 regulatory axis in supporting FAO metabolism and mitochondrial plasticity in metastatic breast cancer cells.

Several lines of evidence reported that DDX3 regulates the translation of mRNAs critical for the cell cycle, DNA repair, and stress response, which makes DDX3 a crucial factor in tumor progression [47,[52], [53], [54]]. DDX3 was found to affect the stability and translation of CDK1 mRNA, thereby regulating CDK1's activity [55]. DDX3 also impacts the translation of cyclins or proteins that modulate CDK1's activity, which mediates the G_2_/M transition [56]. In breast cancer cells, DDX3 is overexpressed and promotes tumor cell proliferation [57]. Inhibition of DDX3 resulted in a global delay in cell cycle progression [55]. Furthermore, DDX3 might form complexes with CDK1 under certain conditions to synergistically modulate the cell cycle [47,55]. Indeed, our research showed for the first time that DDX3 recruits CDK1 to DRP1, which promotes DRP1 phosphorylation by CDK1. DDX3 overexpression might facilitate CDK1 phosphorylating activity on DRP1. Importantly, DDX3 expression did not alter CDK1 levels, while DDX3 impacted CDK1-mediated DRP1 phosphorylation at S616, indicating that DDX3 might dominantly mediate CDK1 activity (Fig. 6). Notably, DDX3's activity was reportedly regulated by protein-protein interactions (PPIs) and post-translational modifications (PTMs). DDX3 phosphorylation was demonstrated to modulate several biological processes including cell mitosis and antiviral innate immunity [55,[58], [59], [60]]. Based on our data, CDK1 was associated with both DRP1 and DDX3 upon lipid stimulation. RO3306 diminished the increase in pDRP1^S616^ levels induced by DDX3 overexpression. We suggest that pDRP1^S616^ reduction might not only be due to decreased CDK1-mediated DRP1 phosphorylation. An interplay between DDX3 and DRP1 might exist in mitochondria, where CDK1 can phosphorylate DDX3 and affect its stability, activity, or even functionality. Consequently, this crosstalk could form a positive feedback loop in response to metabolic stimuli. CDK1 might regulate DDX3's activity to recruit DRP1 in mitochondria. Intriguingly, several studies supported our notion that DDX3 might be a substrate of CDK1 [54,58,61], and CDK1 was reported to coordinate mitochondrial respiration for cell-cycle G2/M progression [62]. Therefore, it would be worthwhile to further identify the interplay between DDX3 and phosphorylation regulation by CDK1 in mitochondria.

Metabolic reprogramming is a determining cancer hallmark during metastasis [19,63]. Regarding the importance of mitochondrial metabolism in tumor progression, dysregulated mitochondrial dynamics were observed in metastasizing tumor cells in various cancer types including breast and colon cancers [64,65]. Indeed, our lipid profiling showed that most lipid subclasses were significantly enriched in 4T1-LM cells (Fig. 1). Additionally, the migratory ability of LM cells was more susceptible to FAO inhibition rather than to other metabolic suppression (Fig. 1), indicating that metastatic breast cancer cells preferentially utilize FAO to meet energy demands. Meanwhile, the process was accompanied with increased mitochondrial fission (Fig. 2). Based on our previous findings of DDX3's regulation of mitochondrial homeostasis [31], we identified DDX3-interacting mitochondrial partners upon metabolic stress stimulation by an IP-MS analysis (Fig. 5), including DRP1 and the mitophagy regulator, STOML2. Similar to our results, one study found by an IP-MS analysis that DDX3 was associated with major components of the cellular stress response and mitochondrial protein quality control including p97/VCP/Cdc48 and heat shock protein 78 (HSP78), which are known to selectively degrade damaged mitochondrial proteins [41]. These findings suggest that DDX3 regulates mitochondrial dynamics to overcome cellular stress. Interestingly, our LC-MS data showed that increased DDX3 interactions with FAO-related proteins were clearly observed upon CCCP induction (Fig. 5), which linked DDX3 and FAO regulation. Thus, it is worth investigating their interplay in the future.

Metastatic breast cancer cells display higher FAO activity compared to their primary tumor counterparts (Fig. 1, Fig. 2), which may endow them with greater metabolic flexibility and resistance to lipid-induced stress. Our lipidomic profiling revealed that mitochondrial phospholipids were more abundant in LM cells, a feature associated with enhanced mitochondrial functions and resistance to apoptosis [66]. In addition, the elevated level of ceramides (Cer) in LM cells further supports the notion that metastatic cells are intrinsically adapted to lipid-induced toxicity (Fig. 1). Beyond its role in energy production, active FAO also contributes to redox homeostasis by generating reducing equivalents and promoting the expression of antioxidant genes, including those regulated by NRF2 [67]. In this study, we found that DDX3 plays a pivotal role in sustaining FAO proficiency. Mechanistically, DDX3 facilitates mitochondrial fission via the CDK1–DRP1 signaling axis, thereby enhancing lipid catabolism. This regulatory function becomes particularly crucial under saturated fatty acid exposure. Although PA can induce lipotoxicity through ceramide accumulation and oxidative stress, LM cells appear more tolerant to PA-induced damage, while the effect is diminished when DDX3 is depleted (Fig. 3, Fig. 4). Notably, both pharmacological inhibition of FAO and DDX3 knockdown sensitized LM cells to PA, indicating that DDX3-driven FAO activity serves a detoxifying role. On the other hand, OA, a monounsaturated fatty acid, is more efficiently metabolized through FAO. The differential OA utilization observed between LM and PT cells, as well as between control and DDX3-depleted breast cancer cells, further underscores the importance of DDX3 in maintaining FAO capacity and supporting metabolic adaptation under lipid stress.

Stem-like tumor cells and latent metastatic cancer cells were reported to exhibit highly fragmented mitochondrial networks, which linked mitochondrial fission to cancer stemness [68]. Herein, we found that metastatic tumor cells showed a more-fragmented network, and we demonstrated that DDX3-mediated mitochondrial fission facilitated FAO utilization (Fig. 7). Conversely, inhibition of FAO or DDX3 had a greater impact on metastatic tumor cells, supporting the notion that the DDX3-mediated metabolic shift to FAO is indispensable for tumor metastasis (Fig. 1, Fig. 3). In line with our findings, targeting the mitochondrial fission regulator protein, DRP1, via genetic depletion, and pharmacological inhibition, using Mdivi-1 abolished brain tumor initiating cells [69], impaired FAO and thus reduced breast cancer brain metastases [11], suggesting that DRP1-mediated mitochondrial plasticity is essential for tumor progression. Similarly, DRP1 activation promoted FA-induced metabolic reprogramming to potentiate the Wnt signaling pathway, in which β-catenin protein acetylation and activation by FA-derived Ac-CoA stimuli [70], indicating that mitochondrial fission facilitated lipid metabolism to promote Wnt signaling at a post-translational level. In addition, inhibiting JAK/STAT3 blocked self-renewal of breast cancer stem cells and expressions of diverse lipid metabolic genes, including FAO-related genes [71]. Suppression of *de novo* lipogenesis via FA synthase (FASN) inhibition was shown to attenuate CD44 expression, possibly through its palmitoylation [[72], [73], [74]], highlighting the association between lipid metabolism and cancer stemness.

Emerging evidence underscores the oncogenic role of DDX3 in cancer progression and therapeutic resistance [57,75,76]. Several studies have demonstrated that elevated DDX3 expression is significantly associated with poor prognosis in breast cancer patients, including shorter overall survival and increased metastatic risk [32]. Our previous work further showed that DDX3 is particularly enriched in TNBC, a highly aggressive subtype with limited therapeutic options [31,77], while pharmacological inhibition of DDX3 significantly suppressed malignant progression. These observations support the notion that DDX3 contributes to tumor aggressiveness and may serve as a potential prognostic marker. In addition to its prognostic significance, DDX3 also appears to influence therapeutic responsiveness. DDX3 knockdown has been shown to sensitize TNBC cells to chemotherapeutic agents and increase radiosensitivity in breast cancer models [31,42]. Combination of DDX3 and PARP inhibition has also reported to enhance sensitivity in BRCA1-proficient breast cancer [78]. Besides, DDX3 degradation has been shown to suppress TNBC progression and enhance chemosensitivity [37]. Moreover, recent findings revealed that DDX3 inhibition activates a tumor-intrinsic type I interferon response via cytosolic dsRNA sensing and reduces surface PD-L1 levels, which may enhance antitumor immunity and improve the efficacy of immune checkpoint blockade [60,79]. These findings collectively suggest that DDX3 plays a dual role in promoting cancer progression and conferring therapeutic resistance. In this context, our present study provides new insights by demonstrating that DDX3 promotes mitochondrial plasticity and fatty acid oxidation to support the aggressiveness of breast cancer cells, such as stemness property and metastatic potential (Fig. 7). Given its involvement in both metabolic adaptation and immune modulation, targeting DDX3 may offer a promising strategy for combination therapies aimed at overcoming the dual challenges of metastasis and therapeutic resistance in breast cancer.

To bridge our mechanistic findings with future therapeutic directions, recent advances in RNA-based therapeutics have highlighted the ability of engineered RNA molecules to selectively modulate disease-driving pathways with high precision [80,81]. While GalNAc-conjugated RNA platforms have achieved significant success in targeting hepatocytes, particularly in siRNA-based therapies, there is still an urgent need to develop delivery technologies that can reach extrahepatic tissues such as tumors [82]. Additionally, improving RNA chemical stability and incorporating transcriptomic and epitranscriptomic profiling will be critical for enabling personalized therapeutic design [83]. Alongside synthetic RNA approaches, growing evidence has revealed the diverse regulatory roles of endogenous RNA species, including circular RNAs and RNA methylation regulators, in inflammation and metabolic adaptation [[84], [85], [86]]. These non-coding RNAs function as sponges or translatable elements, representing a promising class of biomarkers and therapeutic targets. Furthermore, transcriptomic alterations identified in diseases such as Alzheimer's disease, sepsis, and cancers provide additional support for the relevance of RNA-based strategies in complex disease contexts [87]. Together, these insights underscore the potential of both engineered and endogenous RNA-based interventions in metabolic and mitochondrial dysfunctions associated with cancer and inflammatory diseases.

Collectively, our study demonstrated that DDX3 plays a critical role in regulating mitochondrial plasticity, which supports metabolic flexibility for metastatic breast cancer cells and drives the metabolic shift to FAO. DDX3 collaborates with DRP1/CDK1 to promote mitochondrial fission and aggressiveness. Thus, targeting the DDX3-DRP1-CDK1 axis could produce therapeutic susceptibility to FAO inhibition for treating metastatic breast cancer.

## CRediT authorship contribution statement

**Wen-Jing Hsu:** Writing – original draft, Validation, Methodology, Investigation, Formal analysis, Data curation, Conceptualization. **Ming-Chien Hsu:** Software, Methodology, Investigation, Formal analysis, Data curation, Conceptualization. **Cheng-Ying Chu:** Resources, Methodology. **Yu-Cheng Lee:** Resources, Methodology. **Ching-Chieh Yang:** Resources, Methodology. **Zei-Wei Liu:** Methodology, Data curation. **Chi-Ching Lee:** Resources, Methodology. **Yang-Sen Lin:** Methodology. **Cheng-Wei Lin:** Writing – review & editing, Writing – original draft, Validation, Supervision, Methodology, Investigation, Funding acquisition, Conceptualization.

## Declaration of competing interest

The authors declare that they have no known competing financial interests or personal relationships that could have appeared to influence the work reported in this paper.

## Data Availability

Data will be made available on request.

## References

[1] Riggio A.I., Varley K.E., Welm A.L. (2021). The lingering mysteries of metastatic recurrence in breast cancer. Br. J. Cancer.

[2] Park M., Kim D., Ko S., Kim A., Mo K., Yoon H. (2022). Breast cancer metastasis: mechanisms and therapeutic implications. Int. J. Mol. Sci..

[3] Valastyan S., Weinberg Robert A. (2011). Tumor metastasis: molecular insights and evolving paradigms. Cell.

[4] Bergers G., Fendt S.-M. (2021). The metabolism of cancer cells during metastasis. Nat. Rev. Cancer.

[5] Dai J.Z., Wang Y.J., Chen C.H., Tsai I.L., Chao Y.C., Lin C.W. (2022). YAP dictates mitochondrial redox homeostasis to facilitate obesity-associated breast cancer progression. Adv. Sci. (Weinh.).

[6] Huang K., Han Y., Chen Y., Shen H., Zeng S., Cai C. (2025). Tumor metabolic regulators: key drivers of metabolic reprogramming and the promising targets in cancer therapy. Mol. Cancer.

[7] Zhang Y., Tang J., Jiang C., Yi H., Guang S., Yin G. (2025). Metabolic reprogramming in cancer and senescence. MedComm.

[8] Altea-Manzano P., Decker-Farrell A., Janowitz T., Erez A. (2025). Metabolic interplays between the tumour and the host shape the tumour macroenvironment. Nat. Rev. Cancer.

[9] Xu X., Peng Q., Jiang X., Tan S., Yang Y., Yang W. (2023). Metabolic reprogramming and epigenetic modifications in cancer: from the impacts and mechanisms to the treatment potential. Exp. Mol. Med..

[10] Martínez-Reyes I., Chandel N.S. (2021). Cancer metabolism: looking forward. Nat. Rev. Cancer.

[11] Parida P.K., Marquez-Palencia M., Ghosh S., Khandelwal N., Kim K., Nair V. (2023). Limiting mitochondrial plasticity by targeting DRP1 induces metabolic reprogramming and reduces breast cancer brain metastases. Nat. Cancer.

[12] Cheng X., Geng F., Pan M., Wu X., Zhong Y., Wang C. (2020). Targeting DGAT1 ameliorates glioblastoma by increasing fat catabolism and oxidative stress. Cell Metab..

[13] Pascual G., Avgustinova A., Mejetta S., Martín M., Castellanos A., Attolini C.S.-O. (2017). Targeting metastasis-initiating cells through the fatty acid receptor CD36. Nature.

[14] Yang P., Qin H., Li Y., Xiao A., Zheng E., Zeng H. (2022). CD36-mediated metabolic crosstalk between tumor cells and macrophages affects liver metastasis. Nat. Commun..

[15] Hao J.-W., Wang J., Guo H., Zhao Y.-Y., Sun H.-H., Li Y.-F. (2020). CD36 facilitates fatty acid uptake by dynamic palmitoylation-regulated endocytosis. Nat. Commun..

[16] Zaidi N.E., Shazali N.A.H., Leow T.C., Osman M.A., Ibrahim K., Cheng W.H. (2022). CD36-Fatty acid-mediated metastasis via the bidirectional interactions of cancer cells and macrophages. Cells.

[17] Ramakrishnan G., Terry A.R., Nogueira V., Magdy A., Hay N. (2025). Deletion of AMP-Activated protein kinase impairs metastasis and is rescued by ROS scavenging or ectopic CD36 expression. Cell Rep..

[18] Zhou Q., Cao T., Li F., Zhang M., Li X., Zhao H. (2024). Mitochondria: a new intervention target for tumor invasion and metastasis. Mol. Med..

[19] Porporato P.E., Filigheddu N., Pedro J.M.B.-S., Kroemer G., Galluzzi L. (2018). Mitochondrial metabolism and cancer. Cell Res..

[20] Wang L., Zhang T., Wang L., Cai Y., Zhong X., He X. (2017). Fatty acid synthesis is critical for stem cell pluripotency via promoting mitochondrial fission. EMBO J..

[21] Delaunay S., Pascual G., Feng B., Klann K., Behm M., Hotz-Wagenblatt A. (2022). Mitochondrial RNA modifications shape metabolic plasticity in metastasis. Nature.

[22] Li S., Han S., Zhang Q., Zhu Y., Zhang H., Wang J. (2022). FUNDC2 promotes liver tumorigenesis by inhibiting MFN1-mediated mitochondrial fusion. Nat. Commun..

[23] Adhikary A., Mukherjee A., Banerjee R., Nagotu S. (2023). DRP1: at the crossroads of dysregulated mitochondrial dynamics and altered cell signaling in cancer cells. ACS Omega.

[24] Cribbs J.T., Strack S. (2007). Reversible phosphorylation of Drp1 by cyclic AMP-Dependent protein kinase and calcineurin regulates mitochondrial fission and cell death. EMBO Rep..

[25] Ko H.J., Tsai C.Y., Chiou S.J., Lai Y.L., Wang C.H., Cheng J.T. (2021). The phosphorylation status of Drp1-Ser637 by PKA in mitochondrial fission modulates mitophagy via PINK1/Parkin to exert multipolar spindles assembly during mitosis. Biomolecules.

[26] Civenni G., Bosotti R., Timpanaro A., Vàzquez R., Merulla J., Pandit S. (2019). Epigenetic control of mitochondrial fission enables self-renewal of stem-like tumor cells in human prostate cancer. Cell Metab..

[27] Seo B.J., Yoon S.H., Do J.T. (2018). Mitochondrial dynamics in stem cells and differentiation. Int. J. Mol. Sci..

[28] Hong X., Isern J., Campanario S., Perdiguero E., Ramírez-Pardo I., Segalés J. (2022). Mitochondrial dynamics maintain muscle stem cell regenerative competence throughout adult life by regulating metabolism and mitophagy. Cell Stem Cell.

[29] Skoda J., Borankova K., Jansson P.J., Huang M.L.H., Veselska R., Richardson D.R. (2019). Pharmacological targeting of mitochondria in cancer stem cells: an ancient organelle at the crossroad of novel anti-cancer therapies. Pharmacol. Res..

[30] Minarrieta L., Annis M.G., Audet-Delage Y., Kuasne H., Pacis A., St-Louis C. (2024). Mitochondrial elongation impairs breast cancer metastasis. Sci. Adv..

[31] Hsu W.J., Chiang M.C., Chao Y.C., Chang Y.C., Hsu M.C., Chung C.H. (2024). Arginine methylation of DDX3 by PRMT1 mediates mitochondrial homeostasis to promote breast cancer metastasis. Cancer Res..

[32] Bol G.M., Xie M., Raman V. (2015). DDX3, a potential target for cancer treatment. Mol. Cancer.

[33] Lai Y.W., Liu Z.W., Lin M.H., Yang C.C., Chu C.Y., Chung C.H. (2024). Melatonin increases olaparib sensitivity and suppresses cancer-associated fibroblast infiltration via suppressing the LAMB3-CXCL2 axis in TNBC. Pharmacol. Res..

[34] Chung C.-H., Lu K.-Y., Lee W.-C., Hsu W.-J., Lee W.-F., Dai J.-Z. (2020). Fucoidan-based, tumor-activated nanoplatform for overcoming hypoxia and enhancing photodynamic therapy and antitumor immunity. Biomaterials.

[35] Xie M., Vesuna F., Botlagunta M., Bol G.M., Irving A., Bergman Y. (2015). NZ51, a ring-expanded nucleoside analog, inhibits motility and viability of breast cancer cells by targeting the RNA helicase DDX3. Oncotarget.

[36] Heerma van Voss M.R., Schrijver W.A., Ter Hoeve N.D., Hoefnagel L.D., Manson Q.F., van der Wall E. (2017). The prognostic effect of DDX3 upregulation in distant breast cancer metastases. Clin. Exp. Metastasis.

[37] Yao L., Hao Q., Wang M., Chen Y., Cao H., Zhang Q. (2023). KLHL29-mediated DDX3X degradation promotes chemosensitivity by abrogating cell cycle checkpoint in triple-negative breast cancer. Oncogene.

[38] Bordt E.A., Clerc P., Roelofs B.A., Saladino A.J., Tretter L., Adam-Vizi V. (2017). The putative Drp1 inhibitor mdivi-1 is a reversible mitochondrial complex I inhibitor that modulates reactive oxygen species. Dev. Cell.

[39] Deng X., Zhu M., Liu Y., Zhang N., Zhang P., Zeng W. (2025). Suppression of CDK1/Drp1-Mediated mitochondrial fission attenuates dexamethasone-induced extracellular matrix deposition in the trabecular meshwork. Antioxidants Redox Signal..

[40] Xie B., Wang S., Jiang N., Li J.J. (2019). Cyclin B1/CDK1-regulated mitochondrial bioenergetics in cell cycle progression and tumor resistance. Cancer Lett..

[41] Padmanabhan P.K., Zghidi-Abouzid O., Samant M., Dumas C., Aguiar B.G., Estaquier J. (2016). DDX3 DEAD-Box RNA helicase plays a central role in mitochondrial protein quality control in leishmania. Cell Death Dis..

[42] Heerma van Voss M.R., Vesuna F., Bol G.M., Afzal J., Tantravedi S., Bergman Y. (2018). Targeting mitochondrial translation by inhibiting DDX3: a novel radiosensitization strategy for cancer treatment. Oncogene.

[43] Calviello L., Venkataramanan S., Rogowski K.J., Wyler E., Wilkins K., Tejura M. (2021). DDX3 depletion represses translation of mRNAs with complex 5' UTRs. Nucleic Acids Res..

[44] Soto-Rifo R., Ohlmann T. (2013). The role of the DEAD-Box RNA helicase DDX3 in mRNA metabolism. Wiley Interdiscip Rev RNA.

[45] Tsai S.-Y., Lin C.-H., Jiang Y.-T., Huang G.-J., Pi H., Hung H.-Y. (2024). DDX3 is critical for female fertility via translational control in oogenesis. Cell Death Discov..

[46] Lee C.-S., Dias A.P., Jedrychowski M., Patel A.H., Hsu J.L., Reed R. (2008). Human DDX3 functions in translation and interacts with the translation initiation factor eIF3. Nucleic Acids Res..

[47] Ariumi Y. (2014). Multiple functions of DDX3 RNA helicase in gene regulation, tumorigenesis, and viral infection. Front. Genet..

[48] Tu Y.T., Barrientos A. (2015). The human mitochondrial DEAD-box protein DDX28 resides in RNA granules and functions in mitoribosome assembly. Cell Rep..

[49] Antonicka H., Shoubridge Eric A. (2015). Mitochondrial RNA granules are centers for posttranscriptional RNA processing and ribosome biogenesis. Cell Rep..

[50] Otera H., Ishihara N., Mihara K. (2013). New insights into the function and regulation of mitochondrial fission. Biochim. Biophys. Acta Mol. Cell Res..

[51] Zou G.P., Yu C.X., Shi S.L., Li Q.G., Wang X.H., Qu X.H. (2021). Mitochondrial dynamics mediated by DRP1 and MFN2 contributes to cisplatin chemoresistance in human ovarian cancer SKOV3 cells. J. Cancer.

[52] Zhang L., Li X. (2021). DEAD-box RNA helicases in cell cycle control and clinical therapy. Cells.

[53] Chen H.H., Yu H.I., Cho W.C., Tarn W.Y. (2015). DDX3 modulates cell adhesion and motility and cancer cell metastasis via Rac1-mediated signaling pathway. Oncogene.

[54] Choi Y.-J., Lee S.-G. (2012). The DEAD-Box RNA helicase DDX3 interacts with DDX5, co-localizes with it in the cytoplasm during the G2/M phase of the cycle, and affects its shuttling during mRNP export. J. Cell. Biochem..

[55] Heerma van Voss M.R., Kammers K., Vesuna F., Brilliant J., Bergman Y., Tantravedi S. (2018). Global effects of DDX3 inhibition on cell cycle regulation identified by a combined phosphoproteomics and single cell tracking approach. Transl. Oncol..

[56] Lai M.C., Chang W.C., Shieh S.Y., Tarn W.Y. (2010). DDX3 regulates cell growth through translational control of cyclin E1. Mol. Cell Biol..

[57] Winnard P.T., Vesuna F., Bol G.M., Gabrielson K.L., Chenevix-Trench G., ter Hoeve N.D. (2024). Targeting RNA helicase DDX3X with a small molecule inhibitor for breast cancer bone metastasis treatment. Cancer Lett..

[58] Sekiguchi T., Kurihara Y., Fukumura J. (2007). Phosphorylation of threonine 204 of DEAD-Box RNA helicase DDX3 by cyclin B/cdc2 in vitro. Biochem. Biophys. Res. Commun..

[59] Chen W.-J., Wang W.-T., Tsai T.-Y., Li H.-K., Lee Y.-H.W. (2017). DDX3 localizes to the centrosome and prevents multipolar mitosis by epigenetically and translationally modulating p53 expression. Sci. Rep..

[60] Chen H.-H., Yu H.-I., Chang J.J.-S., Li C.-W., Yang M.-H., Hung M.-C. (2024). DDX3 regulates cancer immune surveillance via 3′ UTR-Mediated cell-surface expression of PD-L1. Cell Rep..

[61] Valverde J.M., Dubra G., Phillips M., Haider A., Elena-Real C., Fournet A. (2023). A cyclin-dependent kinase-mediated phosphorylation switch of disordered protein condensation. Nat. Commun..

[62] Wang Z., Fan M., Candas D., Zhang T.-Q., Qin L., Eldridge A. (2014). Cyclin B1/Cdk1 coordinates mitochondrial respiration for cell-cycle G2/M progression. Dev. Cell.

[63] Mauro-Lizcano M., Di Pisa F., Larrea Murillo L., Sugden C.J., Sotgia F., Lisanti M.P. (2024). High mitochondrial DNA content is a key determinant of stemness, proliferation, cell migration, and cancer metastasis in vivo. Cell Death Dis..

[64] Loureiro R., Mesquita K.A., Magalhães-Novais S., Oliveira P.J., Vega-Naredo I. (2017). Mitochondrial biology in cancer stem cells. Semin. Cancer Biol..

[65] Fan M., Shi Y., Zhao J., Li L. (2023). Cancer stem cell fate determination: mito-nuclear communication. Cell Commun. Signal..

[66] Li Y.-J., Fahrmann J.F., Aftabizadeh M., Zhao Q., Tripathi S.C., Zhang C. (2022). Fatty acid oxidation protects cancer cells from apoptosis by increasing mitochondrial membrane lipids. Cell Rep..

[67] Li S.S., Zhang B., Huang C., Fu Y., Zhao Y., Gong L. (2025). FAO-Fueled OXPHOS and NRF2-mediated stress resilience in MICs drive lymph node metastasis. Proc. Natl. Acad. Sci. U. S. A..

[68] Peiris-Pagès M., Bonuccelli G., Sotgia F., Lisanti M.P. (2018). Mitochondrial fission as a driver of stemness in tumor cells: MDIVI1 inhibits mitochondrial function, cell migration and cancer stem cell (CSC) signalling. Oncotarget.

[69] Xie Q., Wu Q., Horbinski C.M., Flavahan W.A., Yang K., Zhou W. (2015). Mitochondrial control by DRP1 in brain tumor initiating cells. Nat. Neurosci..

[70] Xiong X., Hasani S., Young L.E.A., Rivas D.R., Skaggs A.T., Martinez R. (2022). Activation of Drp1 promotes fatty acids-induced metabolic reprograming to potentiate wnt signaling in Colon cancer. Cell Death Differ..

[71] Wang T., Fahrmann J.F., Lee H., Li Y.-J., Tripathi S.C., Yue C. (2018). JAK/STAT3-Regulated fatty acid β-Oxidation is critical for breast cancer stem cell self-renewal and chemoresistance. Cell Metab..

[72] Zaytseva Y.Y., Rychahou P.G., Gulhati P., Elliott V.A., Mustain W.C., O'Connor K. (2012). Inhibition of fatty acid synthase attenuates CD44-associated signaling and reduces metastasis in colorectal cancer. Cancer Res..

[73] Singh M.K., Han S., Kim S., Kang I. (2024). Targeting lipid metabolism in cancer stem cells for anticancer treatment. Int. J. Mol. Sci..

[74] Zhu Y., Chen S., Su H., Meng Y., Zang C., Ning P. (2025). CPT1A-mediated MFF succinylation promotes stemness maintenance in ovarian cancer stem cells. Commun. Biol..

[75] Botlagunta M., Vesuna F., Mironchik Y., Raman A., Lisok A., Winnard P. (2008). Oncogenic role of DDX3 in breast cancer biogenesis. Oncogene.

[76] Bol G.M., Vesuna F., Xie M., Zeng J., Aziz K., Gandhi N. (2015). Targeting DDX3 with a small molecule inhibitor for lung cancer therapy. EMBO Mol. Med..

[77] Dai J.-Z., Hsu W.-J., Lin M.-H., Shueng P.-W., Lee C.-C., Yang C.-C. (2025). YAP-Mediated DDX3X confers resistance to ferroptosis in breast cancer cells by reducing lipid peroxidation. Free Radic. Biol. Med..

[78] Heerma van Voss M.R., Brilliant J.D., Vesuna F., Bol G.M., van der Wall E., van Diest P.J. (2017). Combination treatment using DDX3 and PARP inhibitors induces synthetic lethality in BRCA1-proficient breast cancer. Med. Oncol..

[79] Choi H., Kwon J., Cho M.S., Sun Y., Zheng X., Wang J. (2021). Targeting DDX3X triggers antitumor immunity via a dsRNA-Mediated tumor-intrinsic type I interferon response. Cancer Res..

[80] Wang S., Weissman D., Dong Y. (2025). RNA chemistry and therapeutics. Nat. Rev. Drug Discov..

[81] Huang H.Y.R., Badar S., Said M., Shah S., Bharadwaj H.R., Ramamoorthy K. (2024). The advent of RNA-Based therapeutics for metabolic syndrome and associated conditions: a comprehensive review of the literature. Mol. Biol. Rep..

[82] Qin Z.X., Zuo L., Zeng Z., Ma R., Xie W., Zhu X. (2025). GalNac-siRNA conjugate delivery technology promotes the treatment of typical chronic liver diseases. Expet Opin. Drug Deliv..

[83] Saw P.E., Song E. (2024). Advancements in clinical RNA therapeutics: present developments and prospective outlooks. Cell Rep. Med..

[84] Saaoud F., Drummer I.V.C., Shao Y., Sun Y., Lu Y., Xu K. (2021). Circular RNAs are a novel type of non-coding RNAs in ROS regulation, cardiovascular metabolic inflammations and cancers. Pharmacol. Ther..

[85] Zhang Y., Geng X., Xu J., Li Q., Hao L., Zeng Z. (2021). Identification and characterization of N6-methyladenosine modification of circRNAs in glioblastoma. J. Cell Mol. Med..

[86] Liu M., Xu K., Saaoud F., Shao Y., Zhang R., Lu Y. (2022). 29 m(6)A-RNA methylation (epitranscriptomic) regulators are regulated in 41 diseases including atherosclerosis and tumors potentially via ROS regulation - 102 transcriptomic dataset analyses. J Immunol Res.

[87] Kokkinaki D., Hoffman M., Kalliora C., Kyriazis I.D., Maning J., Lucchese A.M. (2019). Chemically synthesized secoisolariciresinol diglucoside (LGM2605) improves mitochondrial function in cardiac myocytes and alleviates septic cardiomyopathy. J. Mol. Cell. Cardiol..

