# Supplementary material for: Metastatic breast cancer cells are vulnerable to fatty acid oxidation inhibition through DDX3-DRP1-mediated mitochondrial plasticity
Source: Redox Biol. 2025 Aug 26;86:103845. doi: 10.1016/j.redox.2025.103845 (PMC12410181; doi:10.1016/j.redox.2025.103845)
Supplement: Multimedia component 1 [file mmc1.docx]

**Metastatic breast cancer cells are** **vulnerable to fatty acid oxidation inhibition through DDX3-DRP1-mediated mitochondrial plasticity**

**AUTHORS:** Wen-Jing Hsu^1,2*^, Ming-Chien Hsu^1,2*^, Cheng-Ying Chu^3^, Yu-Cheng Lee^1^, Ching-Chieh Yang^4,5,6^, Zei-Wei Liu^1,2^, Chi-Ching Lee^7^, Yang-Sen Lin^2^, Cheng-Wei Lin^1,2,8#^

**AFFILIATIONS:**

*^1^Graduate Institute of Medical Sciences, College of Medicine, Taipei Medical University, Taipei, Taiwan.*

*^2^Department of Biochemistry and Molecular Cell Biology, School of Medicine, College of Medicine, Taipei Medical University, Taipei, Taiwan.*

*^3^CRISPR Gene Targeting Core Lab, Taipei Medical University, Taipei, Taiwan.*

*^4^Department of Radiation Oncology, Chi Mei Medical Center, Tainan, Taiwan.*

*^5^Department of Pharmacy, Chia-Nan University of Pharmacy and Science, Tainan, Taiwan.*

*^6^School of Medicine, College of Medicine, National Sun Yat-sen University, Kaohsiung, Taiwan.*

*^7^Department of Food Engineering, Faculty of Engineering and Natural Sciences, Istanbul Sabahattin Zaim University, Istanbul, Turkey.*

*^8^Drug Development and Value Creation Research Center, Kaohsiung Medical University, Kaohsiung, Taiwan.*

* These authors contributed equally to this work.

^#^**Corresponding author:** Cheng-Wei Lin; Department of Biochemistry and Molecular Biology, Taipei Medical University, 250 Wu-Xing Street, Taipei 11031, Taiwan. Email: cwlin@tmu.edu.tw; Phone: 886-2-27361661 ext. 3160; Fax: 886-2-27356689.

**Supplementary figures**

**
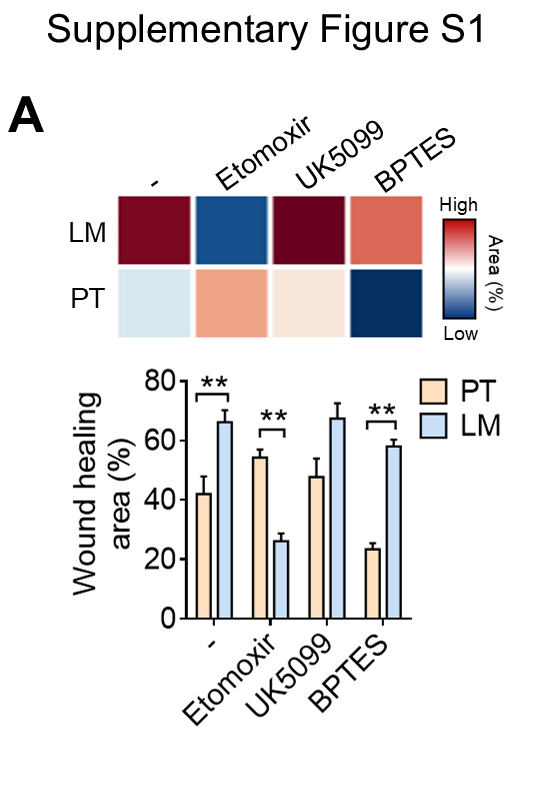
**

**Supplementary figure S1. LM cells are more dependent on FAO to sustain aggressiveness.** (A) Wound-healing assay assessing the migratory capacities of 4T1-PT and 4T1-LM cells upon treatment with the indicated inhibitors. * *p*<0.05, ** *p*<0.01 by an unpaired two-tailed *t*-test.

**
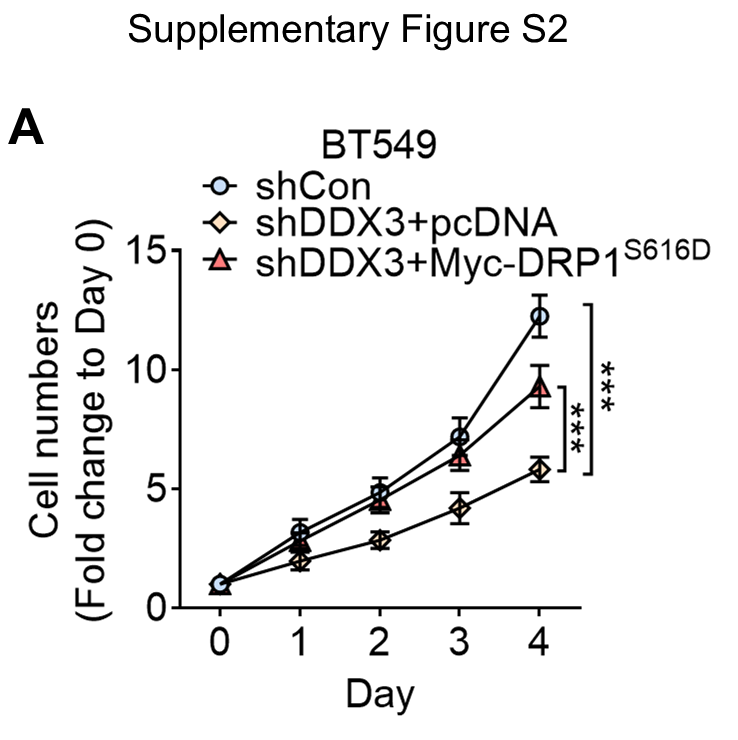
**

**Supplementary figure S2. DDX3 promotes DRP1-mediated DRP1 phosphorylation at serine 616 to regulate cell proliferation.** (A) Cell proliferation assay estimating the cell counts in BT549 shCon, shDDX3+pcDNA and shDDX3+DRP1^S616D^ cells during four days. *** *p*<0.001 by an unpaired two-tailed *t*-test.


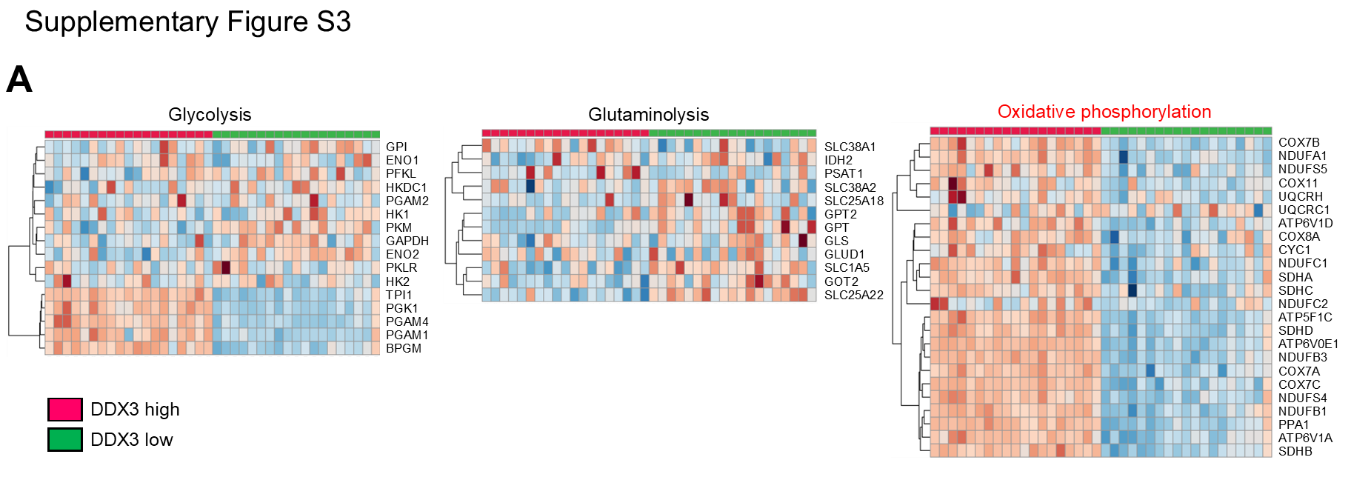


**Supplementary figure S3. Upregulation of DDX3 promotes FAO.** (A) Heatmap showing the correlation between DDX3 and glycolysis-, glutaminolysis- and OXPHOS-related gene expressions in breast cancer patients by analyzing the METABRIC database.

**
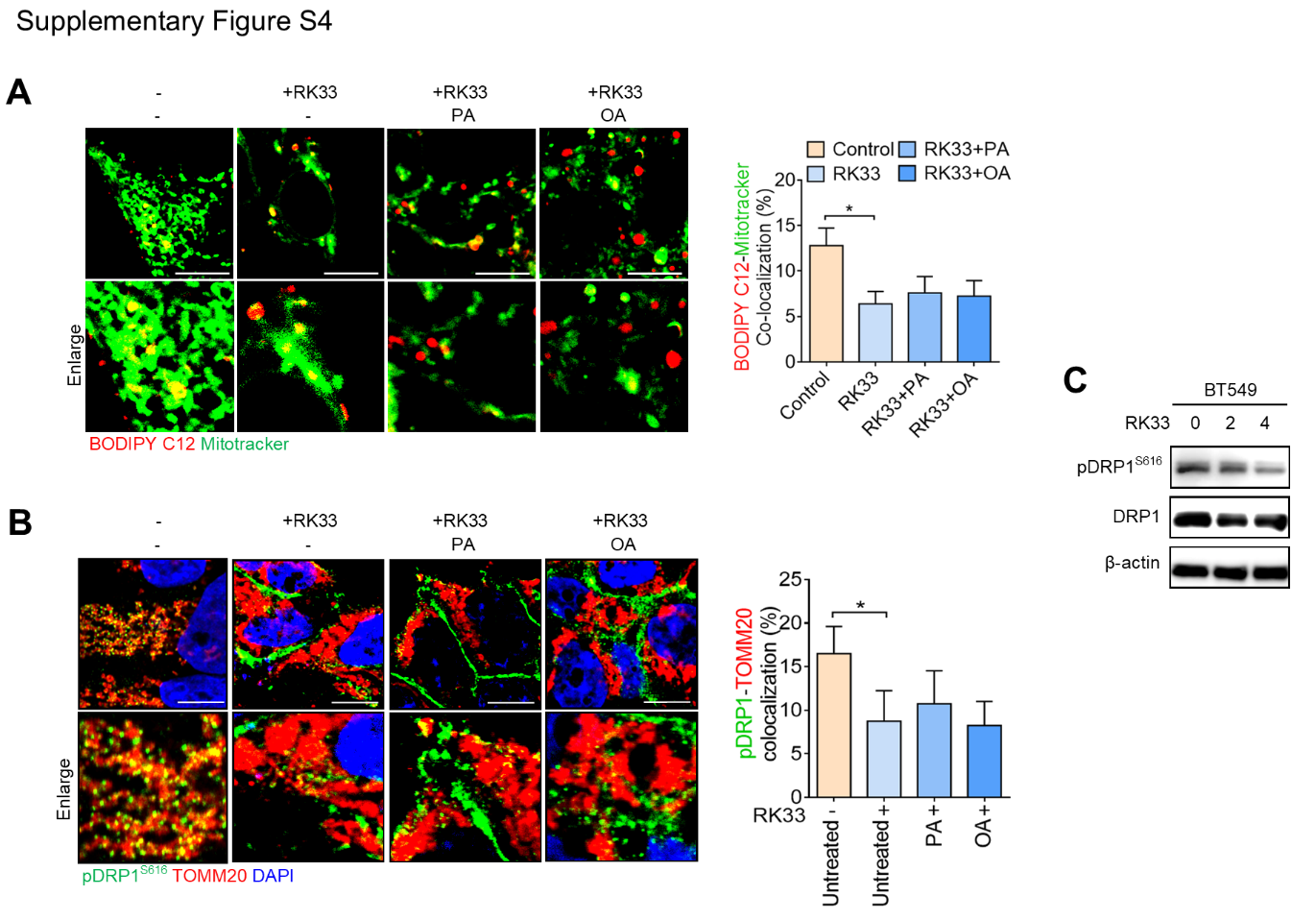

Supplementary figure S4. Pharmacological inhibition of DDX3 impairs lipid-induced mitochondrial fatty acid colocalization and DRP1 activation.** (A) Confocal images showing the colocalization of BODIPY C12 with mitochondria in BT549 cells treated with RK-33, with or without palmitic acid (PA) or oleic acid (OA) stimulation. Quantification of BODIPY C12/MitoTracker colocalization is shown. Scale bars, 10 μm. (B) Confocal immunofluorescence images showing pDRP1^S616^ and TOMM20 in BT549 cells under the indicated conditions. Quantification of pDRP1^S616^/TOMM20 colocalization is shown. Scale bars, 10 μm. (C) Immunoblot analysis of pDRP1 S616 and total DRP1 levels in BT549 cells treated with increasing concentrations of RK-33 (0, 2, or 4 μM).


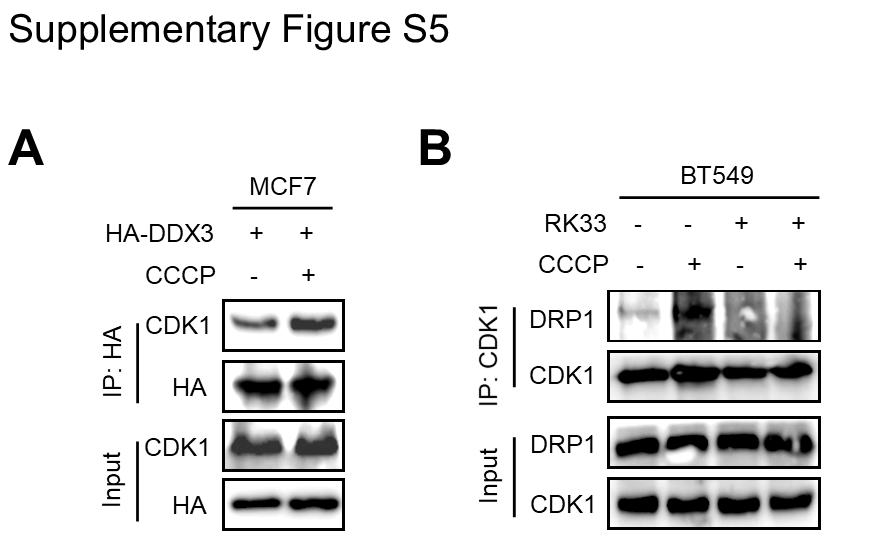


**Supplementary figure S5. Association of DDX3 with DRP1 and CDK1 mediates mitochondrial fission.** (A) Co-IP assay detecting the association between HA-DDX3 and CDK1 upon CCCP induction. (B) Co-IP assay detecting interaction of DRP1 with CDK1 with or without RK33 upon CCCP induction.
